# What is the impact of dexamethasone on postoperative pain in adults undergoing general anaesthesia for elective abdominal surgery: a systematic review and meta-analysis

**DOI:** 10.1186/s13741-022-00243-6

**Published:** 2022-03-24

**Authors:** C. Mitchell, S. J. Cheuk, C. M. O’Donnell, S. Bampoe, D. Walker

**Affiliations:** 1 Department of Anaesthesia, Ulster Hospital, Dundonald, Belfast, Northern Ireland; 2 Department of Anaesthesia, Royal Belfast Hospital for Sick Children, Royal Group of Hospitals, Belfast, Northern Ireland; 3 Department of Anaesthesia, Royal Victoria Hospital, Royal Group of Hospitals, Belfast, Northern Ireland; 4 UCL Centre for Perioperative Medicine, University College London, London, UK

**Keywords:** Dexamethasone, Postoperative pain, Abdominal surgery

## Abstract

**Background:**

Previous meta-analysis of heterogeneous surgical cohorts demonstrated reduction in postoperative pain with perioperative intravenous dexamethasone, but none have addressed adults undergoing elective abdominal surgery. The aim of this study was to determine the impact of intravenous perioperative dexamethasone on postoperative pain in adults undergoing elective abdominal surgery under general anaesthesia.

**Methods:**

This review was prospectively registered on the international prospective register of systematic reviews (CRD42020176202). Electronic databases Medical Analysis and Retrieval System Online (MEDLINE), Exerpta Medica Database (EMBASE), (CINAHL) Cumulative Index to Nursing and Allied Health Literature (CINAHL), Cochrane Central Register of Controlled Trials (CENTRAL), Web of Science and trial registries were searched to January 28 2021 for randomised controlled trials, comparing dexamethasone to placebo or alternative antiemetic, that reported pain. The primary outcome was pain score, and secondary outcomes were time to first analgesia, opioid requirements and time to post-anaesthesia care unit (PACU) discharge.

**Results:**

Fifty-two studies (5768 participants) were included in the meta-analysis. Pain scores ≤ 4 hour (h) were reduced in patients who received dexamethasone at rest (mean difference (MD), − 0.54, 95% confidence interval (CI) − 0.72 to − 0.35, *I*^2^ = 81%) and on movement (MD − 0.42, 95% CI − 0.62 to − 0.22, *I*^2^ = 35). In the dexamethasone group, 4–24 h pain scores were less at rest (MD − 0.31, 95% CI − 0.47 to − 0.14, *I*^2^ = 96) and on movement (MD − 0.26, 95% CI − 0.39 to − 0.13, *I*^2^ = 29) and pain scores ≥ 24 h were reduced at rest (MD − 0.38, 95% CI − 0.52 to − 0.24, *I*^2^ = 88) and on movement (MD − 0.38, 95% CI − 0.65 to − 0.11, *I*^2^ = 71). Time to first analgesia (minutes) was increased (MD 22.92, 95% CI 11.09 to 34.75, *I*^2^ = 98), opioid requirements (mg oral morphine) decreased (MD − 6.66, 95% CI − 9.38 to − 3.93, *I*^2^ = 88) and no difference in time to PACU discharge (MD − 3.82, 95% CI − 10.87 to 3.23, *I*^2^ = 59%).

**Conclusions:**

Patients receiving dexamethasone had reduced pain scores, postoperative opioid requirements and longer time to first analgesia. Dexamethasone is an effective analgesic adjunct for patients undergoing abdominal surgery.

**Supplementary Information:**

The online version contains supplementary material available at 10.1186/s13741-022-00243-6.

## Background

Pain is a common postoperative problem and can be associated with physical and psychological sequelae. Glucocorticoids can modify the stress response and reduce inflammation. Dexamethasone, a commonly used antiemetic, interferes with the cyclooxygenase and lipoxygenase pathways through phospholipase inhibition and has been proposed to modulate postoperative pain in surgical patients (Moore, 2018).

Two reviews, Waldron et al. and De Oliveira et al., established a reduction in postoperative pain from a single perioperative dose of dexamethasone in heterogeneous surgical cohorts with debated clinical significance (Moore, 2018; De Oliveira Jr. et al., 2011a; Waldron et al., 2013). Additionally, they demonstrated dexamethasone’s opioid-sparing effects but produced conflicting conclusions regarding the dose-response relationship (De Oliveira Jr. et al., 2011a; Waldron et al., 2013). Therefore, the analgesic benefit of glucocorticoids in abdominal surgery remains unclear (Ahn et al., 2011; Holte & Kehlet, 2002). Waldron et al. excluded patients who received intrathecal or epidural local anaesthetics or opioids yet regional anaesthesia plays a key role in opioid-sparing analgesia for major abdominal surgery (Waldron et al., 2013). Furthermore, patients who received multiple doses of dexamethasone were excluded potentially limiting their clinical significance considering the surgical stress response extends beyond the period of surgery.

Given the exclusion criteria in reviews to date, it is unclear if any benefit demonstrated from the use of dexamethasone in heterogenous cohorts can be translated into patients undergoing elective abdominal surgery.

Therefore, the aim of this review is to determine the effect of perioperative dexamethasone on postoperative pain in adults undergoing general anaesthesia for elective abdominal surgery.

## Methods

This study was performed according to a prospectively registered protocol (CRD42020176202) and followed guidance from the preferred reporting items for systematic reviews and meta-analysis (PRISMA) statement (Moher et al., 2009; Liberati et al., 2009; Research NIfH, 2020).

Randomised controlled trials (RCT) of adults, aged 18 or over, who received intravenous perioperative dexamethasone undergoing general anaesthesia alone or in combination with regional anaesthesia with pain as a primary or secondary outcome for elective abdominal surgery were included. Gastrointestinal, gynaecological and urological procedures were included but renal or transplant surgery was excluded. As the intention was to assess the impact of dexamethasone on postoperative pain, minor gynaecological procedures that were not considered to be painful for example diagnostic laparoscopy were excluded (Alexander, 1997). Studies were included if intravenous dexamethasone was given at any time, in any dose, either alone or in combination with other antiemetics with placebo or any combination of antiemetic drugs as the comparator. Other study drugs could be given provided the analgesic effect of dexamethasone could be isolated. The primary outcome of our review was pain scores reported on an 11-point numerical scale (0–10). Secondary outcomes for this study included time to first analgesia, opioid requirements and time to post anaesthesia care unit (PACU) discharge.

### Literature search

Electronic databases Medical Analysis and Retrieval System Online (MEDLINE), Exerpta Medica Database (EMBASE), Cumulative Index to Nursing and Allied Health Literature (CINAHL), Cochrane Central Register of Controlled Trials (CENTRAL) and Web of Science were searched, with no language or date restrictions, for RCTs published up to January 28 2021. When available, a standardised search strategy to identify RCTs was used (Lefebvre et al., 2019) and the full search strategy was published (Research NIfH, 2020) (see Additional file 1). Grey literature and trial registers were searched as pre-specified; however, due to the COVID-19 pandemic, the World Health Organisation (WHO) International Clinical Trials Registry Portal (ICTRP) was temporarily closed to external users and not searched as pre-specified (World Health Organisation, 2020). The reference lists of identified studies and relevant systematic reviews were scanned for additional evidence.

Two authors, (CM and SJC), independently screened unblinded citations, assessed full texts for eligibility, extracted data, recorded on a predetermined data extraction form (see Additional file 2) and assessed bias at outcome level using Cochrane guidance (Sterne et al., 2019). When necessary, a third author (CO’D) mediated any disagreements.

When the specific surgical procedure was not stated and attempts to contact the author failed, we excluded minor painless surgical procedures based on the length of surgery, anaesthetic and surgical technique, length of hospital stay and postoperative analgesic requirements. Pain scores were defined as early (≤ 4 h), intermediate (4–24 h) and late (≥ 24 h) and pain scores presented as a range of times were allocated to the group they most closely corresponded, for example, 0 to 6 h was allocated to the early group. When multiple pain scores were presented for a single time interval, the latest pain score was extracted. Pain scores were assumed to be at rest when this was not stated and converted from a 0–100 to a 0–10 scale as required. Opioids were combined to achieve the total postoperative dose and converted to oral morphine equivalents (see Supplementary Table 1, Additional File 3). Time to first analgesia and PACU discharge were collected in minutes. Authors were successfully contacted for unpublished data or study clarification in seven studies (Bataille et al., 2016; Jo et al., 2012; Sanchez-Ledesma et al., 2002; Ko-Iam et al., 2015; Chen et al., 2020; D'Souza et al., 2011; Kirdak et al., 2008). Data was extracted as mean and standard deviation or converted using verified methods (Higgins & Deeks, 2019; Hozo et al., 2005; Luo et al., 2018; Wan et al., 2014). Studies containing multiple groups were combined into those with dexamethasone, irrespective of dose or timing, and those not containing dexamethasone. When the analgesic effect of dexamethasone could not be isolated, a subset of study data was included to exclude confounding analgesia.

Meta-analysis of outcome data using a random-effects model was performed using *Review Manager* (*(RevMan*) [Computer program]. Version 5.4, The Cochrane Collaboration, 2020) and presented as mean difference (MD) with 95% confidence intervals (CIs). Statistical heterogeneity was assessed using the method proposed by Higgins et al. (*I*^2^ test) (Higgins et al., 2003).

## Results

**Fig. 1:**
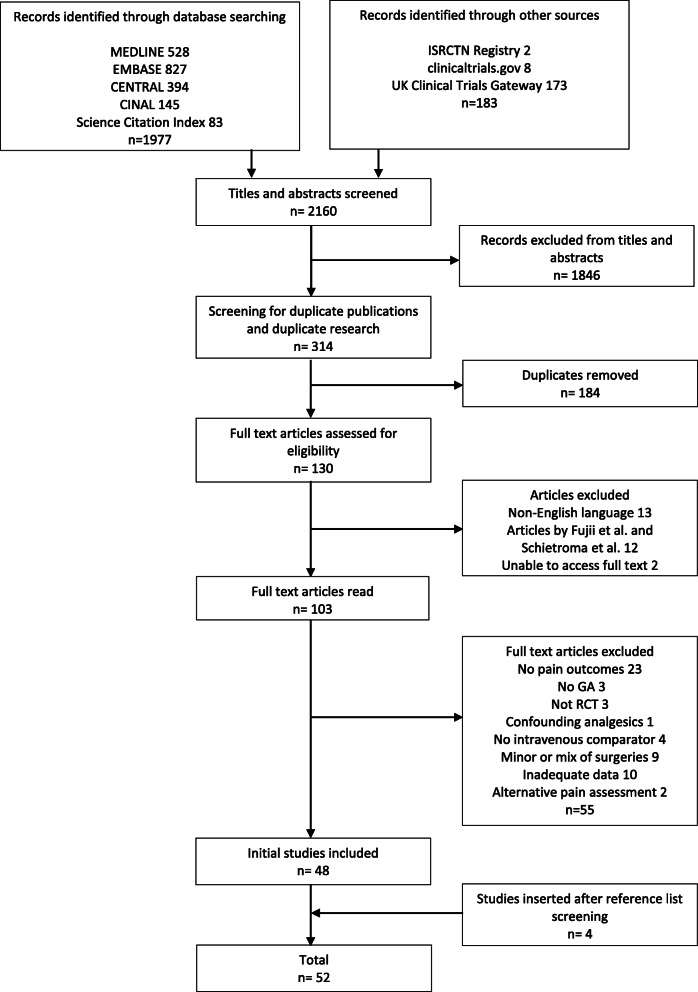
PRISMA flow diagram detailing process of study selection

Database and trial registry searches revealed a total of 2160 citations. Altogether, 1846 irrelevant citations were removed, followed by 184 research and publication duplicates leaving 130 articles for eligibility assessment. Twelve articles by Fujii et al. and Schietroma et al. were excluded due to concerns over research validity and multiple retractions (Rasmussen et al., 2012; Carlisle, 2012; Scott, 2012; Myles et al., 2019). We were unable to obtain two full text articles and 13 non-English articles were removed. One hundred and three articles remained for full text eligibility assessment. Studies failed to meet the inclusion criteria and were excluded for the following reasons; 23 articles reported no pain outcomes, three studies were not RCTs, two studies had mixed surgical cohorts, participants did not receive general anaesthesia in three studies, there was no intravenous comparator in four studies and in one the analgesic effect of dexamethasone could not be isolated. Seven studies with minor surgery were excluded (Abreu et al., 2006; Asadollah et al., 2014; Lee et al., 2003; Ormel et al., 2011; Rajeeva et al., 1998; SS, 2007; Thomas & Jones, 2001). A further 12 studies were excluded; two used an alternative method of pain assessment and 10 presented inadequate data for analysis that we were unable to obtain through contacting the authors. Forty-eight full text articles remained, and four additional studies were included after reference list searching resulting in 52 studies with a total of 5758 participants articles (Fig. 1).

The final included studies are summarised in the characteristics of included studies table (Table 1). All 52 studies were RCTs of adult patients undergoing general anaesthesia for abdominal surgery published in English. The most common dose of dexamethasone used was 8 mg but ranged from 1.25 to 20 mg. Four studies presented the dose of dexamethasone in mg kg^−1^ and were transformed into total doses using the mean study weights or the average weight of an adult at the time and location of the study (De Oliveira Jr. et al., 2011b; Kassim et al., 2018; Lee et al., 2017; Rothenberg et al., 1998; Fryar et al., 2018). No studies administered multiple doses of dexamethasone, but six studies included two or more different doses of dexamethasone (De Oliveira Jr. et al., 2011b; Elhakim et al., 2002; Jokela et al., 2009; Liu et al., 1999; Murphy et al., 2014; Thangaswamy et al., 2010). A further two studies compared the same dose of dexamethasone at different times of administration (Lim et al., 2011; Wang et al., 2000). Opioid doses presented in mg kg^−1^ were converted in a similar manner to dexamethasone (Ko-Iam et al., 2015; Jokela et al., 2009; Pajunen et al., 2012).

**Table 1:** Included studies characteristics, intervention and control groups extracted, primary study outcome and pain outcomes reported

Study 1^**st**^ author year	Country	Procedures	Participants included, no	Participant characteristics	Intervention(s) and control(s)	Primary outcome(s)	Pain outcome(s) reported
Alghanem 2010 (Alghanem et al., 2010)	Jordan	Laparoscopic cholecystectomy	180	ASA ≤ 2 Age 18–70 Mix M/F	I: Intraoperative dexamethasone 8 mg IV C: Ondansetron or saline	PONV	Pain scores
Areeruk 2016 (Areeruk et al., 2016)	Thailand	Gynaecological laparotomy via pfannenstiel incision	49	ASA ≤ 2 Age 18-65	I: Intraoperative dexamethasone 8 mg IV C: Saline	Total morphine consumption	Pain scores Opioid consumption
Badawy 2015 (Badawy & Sakka, 2015)	Egypt	Total abdominal hysterectomy	38	ASA ≤2 Age 40–70	I: Preoperative dexamethasone 8 mg IV + gabapentin 800 mg PO C: Saline + gabapentin	Time to first analgesia	Pain scores Time to first analgesia Opioid consumption
Bataille 2016 (Bataille et al., 2016)	France	Laparoscopic sleeve gastrectomy	117	ASA ≤ 3 Age 18–75 BMI >40 ≥ 2 RFs PONV Mix M/F	I: Intraoperative dexamethasone 4 mg + ondansetron 4 mg IV C: Saline	PONV	Pain scores
Batistaki 2019 (Batistaki et al., 2019)	Greece	Laparoscopic cholecystectomy	44	ASA ≤ 3 Age 18–75 Predominantly female	I: Intraoperative dexamethasone 5 mg C: Saline	Reversal NMB	Pain scores
Benevides 2013 (Benevides et al., 2013)	Brazil	Laparoscopic sleeve gastrectomy	60	ASA 1–3 Age 18+ BMI ≥ 35	I: Intraoperative dexamethasone 8 mg + ondansetron 8 mg IV C: Saline + ondansetron	PONV Rescue antiemetic use	Opioid consumption PACU LOS
Bianchin 2007 (Bianchin et al., 2007)	Italy	Laparoscopic cholecystectomy	73	ASA ≤ 2 Predominantly female	I: Preoperative dexamethasone 8 mg IV C: Saline	PONV	Pain scores
Biligin 2010 (Bilgin et al., 2010)	Turkey	Total abdominal hysterectomy + bilateral salpingoophorectomy	160	ASA ≤ 2 Age 20-60	I: Preoperative dexamethasone 8 mg IV C: Saline or ondansetron or metoclopramide	PONV	Pain scores
Bisgaard 2003 (Bisgaard et al., 2003)	Denmark	Laparoscopic cholecystectomy	80	ASA ≤ 2 Age < 75 Predominantly female	I: Preoperative dexamethasone 8 mg IV C: Saline	Pain Fatigue	Pain Scores Opioid consumption PACU LOS
Coloma 2002 (Coloma et al., 2002)	USA	Laparoscopic cholecystectomy	140	ASA ≤ 2 Predominantly female	I: Intraoperative dexamethasone 4 mg IV C: Saline	Recovery times	Time to first analgesia Opioid consumption PACU LOS
Corcoran 2017 (Corcoran et al., 2017)	Australia	Major laparoscopic gynaecological surgery	31	ASA ≤ 2 Age 18–60 Surgery > 90 min ≥ 1-night stay	I: Intraoperative dexamethasone 4 mg IV C: Saline	Immune response	Pain scores
De Oliveira 2011 (De Oliveira Jr. et al., 2011b)	USA	Laparoscopic gyanecological surgery	106	ASA ≤ 2	I: Preoperative dexamethasone 0.05 or 0.1 mg kg^−1^ IV C: Saline	QoR-40	Pain scores Time to first analgesia Opioid consumption
Elhakim 2002 (Elhakim et al., 2002)	Egypt	Laparoscopic cholecystectomy	180	Predominantly male	I: Preoperative dexamethasone 2 or 4 or 8 or 16 mg IV C: Saline or ondansetron	PONV	Pain scores Time to first analgesia Opioid consumption
Feo 2006 (Feo et al., 2006)	Italy	Laparoscopic cholecystectomy	101	ASA ≤ 3 F>M	I: Preoperative dexamethasone 8mg IV C: Saline	Pain PONV Analgesic and antiemetic requirements	Pain scores
Fukami 2009 (Fukami et al., 2009)	Japan	Laparoscopic cholecystectomy	80	Mix M/F	I: Preoperative dexamethasone 8 mg IV C: Saline	PONV Pain Fatigue Analgesic and antiemetic requirements	Pain scores
Gautam 2008 (Gautam et al., 2008)	Nepal	Laparoscopic cholecystectomy	142	ASA ≤ 2 Age 23–65	I: Preoperative dexamethasone 8 mg IV or dexamethasone 8 mg IV + ondansetron 4 mg C: Ondansetron	PONV	Pain scores Time to first analgesia Opioid consumption
Hammas 2002 (Hammas et al., 2002)	Sweden	Cholecystectomy, Inguinal hernia repair	76	ASA ≤ 2 Predominantly male	I: Intraoperative dexamethasone 4 mg + ondansetron 4 mg + droperidol 1.25 mg + metoclopramide 10 mg IV C: Propofol infusion	PONV	Opioid consumption
Ionescu 2014 (Ionescu et al., 2014)	Romania	Laparoscopic cholecystectomy	42	ASA ≤ 2 Predominantly female	I: Preoperative dexamethasone 4 mg IV C: Saline	Immune response	Opioid consumption
Jo 2012 (Jo et al., 2012)	Korea	Laparoscopic cholecystectomy	120	ASA ≤ 2 Female Age 21–64 BMI < 35	I: Preoperative dexamethasone 8mg IV + saline intraoperative or preoperative dexamethasone 8mg IV + ramosetron 0.3 mg IV intraoperative C: Saline + ramosetron	PONV	Pain scores
Jokela 2009 (Jokela et al., 2009)	Finland	Laparoscopic hysterectomy +/- oophorectomy	120	ASA ≤ 3 BMI < 35	I: Preoperative dexamethasone 5 or 10 or 15mg IV C: Saline	Pain Opioid consumption	Pain scores Time to first analgesia Opioid consumption
Kasagi 2013 (Kasagi et al., 2013)	Japan	Hysterectomy, cystectomy, myomectomy	60	ASA ≤ 2 Age 20–-50 Benign disease	I: Preoperative dexamethasone 8 mg IV C: Droperidol	PONV	Pain scores
Kassim 2018 (Kassim et al., 2018)	Egypt	Laparoscopic gyanecological surgery for infertility	50	ASA ≤ 2 Age 25–35	I: Preoperative dexamethasone 0.1 mg kg^−1^ IV + duloxetine 60 mg PO C: Saline + duloxetine	Pethidine requirements	Pain scores Time to first analgesia Opioid consumption
Ko-iam 2015 (Ko-Iam et al., 2015)	Thailand	Laparoscopic cholecystectomy	100	ASA ≤ 2 Age 18–75 Predominantly female	I: Intraoperative dexamethasone 8 mg + metoclopramide 10 mg IV C: Metoclopramide	PONV	Pain scores Opioid consumption
Kurz 2015 (Kurz et al., 2015)	USA	Colorectal resection	555	Age ≤ 80 Surgery 2–6 h	I: Intraoperative dexamethasone 4 mg IV & 30% oxygen or intraoperative dexamethasone 4 mg IV & 80% oxygen C: Saline + oxygen	Surgical site infection	Pain scores
Lee 2017 (Lee et al., 2017)	Republic of Korea	Laparoscopic cholecystectomy	380	ASA ≤ 2 Age 18–45 Mix M/F	I: Preoperative dexamethasone 5 mg IV C: Saline	Morphine requirements	Pain scores Time to first analgesia Opioid consumption
Lim 2011 (Lim et al., 2011)	Korea	Laparoscopic cholecystectomy	120	ASA ≤ 2 M>F	I: Preoperative dexamethasone 8 mg + intraoperative saline or preoperative saline + intraoperative dexamethasone 8 mg IV C: Saline	Pain	Pain scores Opioid consumption
Liu 1998 (Liu et al., 1998)	Taiwan	Major gynaecological surgery	60	ASA ≤ 2	I: Intraoperative dexamethasone 10 mg C: Saline	PONV Pain	Pain scores Opioid consumption
Liu 1999 (Liu et al., 1999)	Taiwan	Abdominal and radical hysterectomy, myomectomy	150	ASA ≤ 2	I: Preoperative dexamethasone 1.25 or 2.5 or 5 or 10 mg IV C: Saline	PONV	Pain scores Time to first analgesia Opioid consumption
Lopez-Olaondo 1996 (Lopez-Olaondo et al., 1996)	Spain	Major abdominal gynaecological surgery	100	ASA ≤ 2 Age 18–65 45–90 kg	I: Preoperative dexamethasone 8 mg + ondansetron 4 mg IV C: Saline + ondansetron	PONV	Pain scores Opioid consumption
Maddali 2003 (Maddali et al., 2003)	Oman	Laparoscopic gyanecological surgery	120	ASA ≤ 2 Age ≤ 60	I: Preoperative dexamethasone 8 mg + ondansetron 4 mg IV or dexamethasone 8mg + metoclopramide 10 mg C: Saline	PONV	Pain scores
Mathiesen 2009 (Mathiesen et al., 2009)	Denmark	Abdominal hysterectomy +/- salpingoophorectomy	76	ASA ≤ 2 Age 18–75 BMI 18–32	I: Preoperative dexamethasone 8 mg IV+ paracetamol 1g PO+ pregabalin 300 mg PO C: Saline + paracetamol + gabapentin	Morphine consumption	Pain scores Opioid consumption
McKenzie 1997 (McKenzie et al., 1997)	USA	Abdominal or vaginal hysterectomy, laparotomy, anterior and posterior repair	80	ASA ≤ 3 Age 18–65	I: Intraoperative dexamethasone 20 mg IV + ondansetron 4 mg IV C: Saline + ondansetron	PONV	Pain scores Opioid consumption
Murphy 2011 (Murphy et al., 2011)	USA	Laparoscopic cholecystectomy	115	ASA ≤ 3 F>M	I: Preoperative dexamethasone 8 mg IV C: Saline	QoR-40	Pain scores Opioid consumption PACU LOS
Murphy 2014 (Murphy et al., 2014)	USA	Laparoscopic or open hysterectomy	195	ASA ≤ 3 Age 18–80	I: Intraoperative dexamethasone 4 or 8 mg IV C: Saline	Perioperative glucose concentration	Pain scores Opioid consumption PACU LOS
Nesek-Adam 2007 (Nesek-Adam et al., 2007)	Croatia	Laparoscopic cholecystectomy	160	ASA ≤ 2 Predominantly female	I: Intraoperative dexamethasone 8 mg IV + saline or dexamethasone 8 mg + metoclopramide 10 mg C: Saline or saline + metoclopramide	PONV	Pain scores Time to first analgesia
Olajumoke 2013 (Olajumoke et al., 2013)	Nigeria	Total abdominal hysterectomy, myomectomy	96	ASA ≤ 2 Age 18–65	I: Intraoperative dexamethasone 4 mg IV C: Saline	PONV	PACU LOS
Pan 2008 (Pan et al., 2008)	USA	Laparoscopic gynaecological surgery	60	ASA ≤ 2 Age ≥18 ≥ 3 emetic risk factors	I: Intraoperative dexamethasone 8 mg + ondansetron 4 mg IV + ondansetron PO D0,1,2 C: Saline + ondansetron	PONV	Pain scores Opioid consumption PACU LOS
Pauls 2015 (Pauls et al., 2015)	USA	Major vaginal reconstructive surgery	63	ASA ≤ 3	I: Preoperative dexamethasone 8 mg IV C: Saline	Quality of recovery	Pain scores Opioid consumption
Regasa 2020 (Regasa et al., 2020)	Ethiopia	Major gynaecological surgery	96	ASA ≤ 2 Age 18–65	I: Intraoperative dexamethasone 8 mg IV + saline or dexamethasone 8 mg + metoclopramide 10 mg C: metoclopramide	PONV	Opioid consumption
Rothenberg 1998 (Rothenberg et al., 1998)	USA	Laparoscopic gynaecological surgery	95	ASA ≤ 2	I: Intraoperative dexamethasone 0.17 mg kg^−1^ IV C: Droperidol	PONV	Pain scores Opioid consumption PACU LOS
Ryu 2013 (Ryu et al., 2013)	Korea	Laparoscopic cholecystectomy	72	ASA ≤ 2 Age 25–65	I: Intraoperative dexamethasone 8 mg + ramosetron 0.3 mg IV C: Saline + ramosetron	PONV	Pain scores
Sanchez-Ledesma 2002 (Sanchez-Ledesma et al., 2002)	Spain	Hysterectomy, myomectomy/adnexectomy, oncological gynaecological reduction	90	ASA ≤ 2 Age 18–65 45–90 kg	I: Intraoperative dexamethasone 8 mg + droperidol 1.25 mg IV + postoperative droperidol or dexamethasone 8 mg + ondansetron 4 mg and postoperative saline C: Ondansetron + droperidol	PONV	Pain scores Opioid consumption
Sanchez-Rodriquez 2010 (Sanchez-Rodriguez et al., 2010)	Mexico	Laparoscopic cholecystectomy	210	ASA ≤ 2 Age ≤ 80 F>M	I: Preoperative dexamethasone 8 mg IV C: Placebo	PONV Pain Fatigue Additional analgesic and antiemetic drugs	Pain scores
Shrestha 2014 (Shrestha et al., 2014)	Nepal	Laparoscopic cholecystectomy	120	ASA ≤ 2 Age 17–75 Predominantly female	I: Preoperative dexamethasone 8 mg + pheniramine 45.5 mg IV C: Saline	Pain Systemic acute phase response	Pain scores
Sistla 2009 (Sistla et al., 2009)	India	Laparoscopic cholecystectomy	70	Predominantly female	I: Preoperative dexamethasone 8 mg IV C: Saline	Morphine consumption	Pain scores Opioid consumption
Thangaswamy 2010 (Thangaswamy et al., 2010)	India	Total laparoscopic hysterectomy	55	ASA ≤ 2 Age 18–60	I: Preoperative dexamethasone 4 or 8 mg IV C: Saline	Fentanyl consumption	Pain scores Time to first analgesia Opioid consumption
Tolver 2012 (Tolver et al., 2012)	Denmark	Transabdominal preperitoneal groin repair	73	ASA ≤ 2 Age 18–5	I: Preoperative dexamethasone 8 mg IV C: Saline	Pain	Pain scores Opioid consumption
Viriyaroj 2015 (Viriyaroj et al., 2015)	Thailand	Laparoscopic cholecystectomy	80	Predominantly female	I: Preoperative dexamethasone 8 mg IV C: Saline	Pain Analgesic consumption	Pain scores Opioid consumption
Wang 1999 (Wang et al., 1999)	Taiwan	Laparoscopic cholecystectomy	78	ASA ≤ 2 Age 30–55 Predominantly female	I: Preoperative dexamethasone 8 mg IV C: Saline	PONV	Pain scores Opioid consumption
Wang 2000 (Wang et al., 2000)	Taiwan	Total abdominal hysterectomy	120	ASA ≤ 2 Age 35-45	I: Preoperative dexamethasone 10 mg IV + postoperative saline or preoperative saline + postoperative dexamethasone 10 mg IV C: Saline	PONV	Pain scores Opioid consumption
Wu 2009 (Wu et al., 2009)	Taiwan	Anorectal surgery	60	ASA ≤ 2 Predominantly female	I: Preoperative dexamethasone 5 mg IV C: Saline	PONV	Pain scores Opioid consumption
Yuksek 2003 (Yuksek et al., 2003)	Turkey	Laparoscopic gyanecological surgery	60	ASA ≤ 2 19–62	I: Preoperative dexamethasone 8 mg IV C: Saline or ondansetron	PONV	Pain scores Time to first analgesia

*PONV* postoperative nausea and vomiting, *NMB* neuromuscular blockade, *PACU LOS* post-anaesthesia care unit length of stay

Dexamethasone was directly compared to placebo in 27 studies (Areeruk et al., 2016; Batistaki et al., 2019; Bianchin et al., 2007; Bisgaard et al., 2003; Coloma et al., 2002; Corcoran et al., 2017; De Oliveira Jr. et al., 2011b; Feo et al., 2006; Fukami et al., 2009; Ionescu et al., 2014; Jokela et al., 2009; Lee et al., 2017; Lim et al., 2011; Liu et al., 1998; Liu et al., 1999; Murphy et al., 2011; Murphy et al., 2014; Olajumoke et al., 2013; Pauls et al., 2015; Sanchez-Rodriguez et al., 2010; Sistla et al., 2009; Thangaswamy et al., 2010; Tolver et al., 2012; Viriyaroj et al., 2015; Wang et al., 1999; Wang et al., 2000; Wu et al., 2009) with a further four comparing dexamethasone to placebo or another antiemetic (Alghanem et al., 2010; Bilgin et al., 2010; Elhakim et al., 2002; Yuksek et al., 2003). Intravenous anti-emetic drugs were included in the intervention or control groups in 17 studies (Bataille et al., 2016; Jo et al., 2012; Sanchez-Ledesma et al., 2002; Ko-Iam et al., 2015; Benevides et al., 2013; Gautam et al., 2008; Hammas et al., 2002; Kasagi et al., 2013; Lopez-Olaondo et al., 1996; Maddali et al., 2003; McKenzie et al., 1997; Nesek-Adam et al., 2007; Pan et al., 2008; Regasa et al., 2020; Rothenberg et al., 1998; Ryu et al., 2013; Shrestha et al., 2014). One study compared dexamethasone with an intraoperative and postoperative propofol infusion (Hammas et al., 2002). Four studies included additional study drugs, but groups were extracted to ensure the analgesic effect of dexamethasone was isolated (Badawy & Sakka, 2015; Kassim et al., 2018; Kurz et al., 2015; Mathiesen et al., 2009).

The timing of dexamethasone varied from 2 h preoperatively to immediately after extubation (Kassim et al., 2018; Thangaswamy et al., 2010; Wang et al., 2000). Dexamethasone was most frequently given preoperatively (Jo et al., 2012; Badawy & Sakka, 2015; Bianchin et al., 2007; Bilgin et al., 2010; Bisgaard et al., 2003; De Oliveira Jr. et al., 2011b; Elhakim et al., 2002; Feo et al., 2006; Fukami et al., 2009; Gautam et al., 2008; Ionescu et al., 2014; Jokela et al., 2009; Kasagi et al., 2013; Kassim et al., 2018; Lee et al., 2017; Lim et al., 2011; Liu et al., 1999; Lopez-Olaondo et al., 1996; Maddali et al., 2003; Mathiesen et al., 2009; Murphy et al., 2011; Pauls et al., 2015; Regasa et al., 2020; Sanchez-Rodriguez et al., 2010; Shrestha et al., 2014; Sistla et al., 2009; Thangaswamy et al., 2010; Tolver et al., 2012; Viriyaroj et al., 2015; Wang et al., 1999; Wang et al., 2000; Wu et al., 2009; Yuksek et al., 2003), but when administered intraoperatively this was more commonly postinduction pre-incision (Bataille et al., 2016; Sanchez-Ledesma et al., 2002; Alghanem et al., 2010; Areeruk et al., 2016; Batistaki et al., 2019; Benevides et al., 2013; Coloma et al., 2002; Corcoran et al., 2017; Hammas et al., 2002; Kurz et al., 2015; Liu et al., 1998; McKenzie et al., 1997; Murphy et al., 2014; Nesek-Adam et al., 2007; Olajumoke et al., 2013; Pan et al., 2008; Rothenberg et al., 1998; Ryu et al., 2013) than during the surgical procedure (Ko-Iam et al., 2015; Lim et al., 2011). In one study, dexamethasone was given immediately post extubation (Wang et al., 2000).

The primary outcome was most commonly related to postoperative nausea and vomiting (PONV) in 26 studies (Bataille et al., 2016; Jo et al., 2012; Sanchez-Ledesma et al., 2002; Ko-Iam et al., 2015; Alghanem et al., 2010; Benevides et al., 2013; Bianchin et al., 2007; Bilgin et al., 2010; Elhakim et al., 2002; Gautam et al., 2008; Hammas et al., 2002; Kasagi et al., 2013; Liu et al., 1999; Lopez-Olaondo et al., 1996; Maddali et al., 2003; McKenzie et al., 1997; Nesek-Adam et al., 2007; Olajumoke et al., 2013; Pan et al., 2008; Regasa et al., 2020; Rothenberg et al., 1998; Ryu et al., 2013; Wang et al., 1999; Wang et al., 2000; Wu et al., 2009; Yuksek et al., 2003). Pain outcomes were the primary outcome in 11 studies (Areeruk et al., 2016; Badawy & Sakka, 2015; Jokela et al., 2009; Kassim et al., 2018; Lee et al., 2017; Lim et al., 2011; Mathiesen et al., 2009; Sistla et al., 2009; Thangaswamy et al., 2010; Tolver et al., 2012; Viriyaroj et al., 2015) and was a joint primary outcome in a further six studies (Bisgaard et al., 2003; Feo et al., 2006; Fukami et al., 2009; Liu et al., 1998; Sanchez-Rodriguez et al., 2010; Shrestha et al., 2014). The primary outcome was quality or timing of recovery in four studies (Coloma et al., 2002; De Oliveira Jr. et al., 2011b; Murphy et al., 2011; Pauls et al., 2015), the immune or stress response in two studies (Corcoran et al., 2017; Ionescu et al., 2014), surgical site infection in one study (Kurz et al., 2015), perioperative glucose concentration in one study (Murphy et al., 2014) and reversal of neuromuscular blockade in one study (Batistaki et al., 2019). In general, study outcomes were poorly documented with 25 studies not specifically stating study outcome (Jo et al., 2012; Ko-Iam et al., 2015; Alghanem et al., 2010; Bianchin et al., 2007; Bilgin et al., 2010; Coloma et al., 2002; Corcoran et al., 2017; Elhakim et al., 2002; Gautam et al., 2008; Hammas et al., 2002; Ionescu et al., 2014; Jokela et al., 2009; Lim et al., 2011; Liu et al., 1998; Liu et al., 1999; Nesek-Adam et al., 2007; Olajumoke et al., 2013; Regasa et al., 2020; Rothenberg et al., 1998; Ryu et al., 2013; Shrestha et al., 2014; Sistla et al., 2009; Wang et al., 1999; Wang et al., 2000; Yuksek et al., 2003), seven studies documenting primary outcome only (Sanchez-Ledesma et al., 2002; Badawy & Sakka, 2015; Bisgaard et al., 2003; De Oliveira Jr. et al., 2011b; Maddali et al., 2003; Pan et al., 2008; Wu et al., 2009) and ambiguity over primary or secondary outcomes in a further five studies (Feo et al., 2006; Fukami et al., 2009; Kasagi et al., 2013; Sanchez-Rodriguez et al., 2010; Viriyaroj et al., 2015).

Pain was presented on an 11-point numerical scale in the majority of studies and divided by 10 when presented as 0–100 (Jo et al., 2012; Lee et al., 2017; Mathiesen et al., 2009; Murphy et al., 2011; Murphy et al., 2014; Ryu et al., 2013; Thangaswamy et al., 2010; Tolver et al., 2012). Six studies did not report pain scores (Benevides et al., 2013; Coloma et al., 2002; Hammas et al., 2002; Ionescu et al., 2014; Olajumoke et al., 2013; Regasa et al., 2020) and we were unable to extract pain scores in a further four studies (Bisgaard et al., 2003; Kassim et al., 2018; Liu et al., 1998; Lopez-Olaondo et al., 1996). The pain outcomes extracted from each study are presented in Table 2.

**Table 2:** Pain outcomes extracted from each study

Study year	Pain scores								
	Early rest	Early movement	Intermediate rest	Intermediate movement	Late rest	Late movement	Time to first analgesia	Opioid consumption	PACU LOS
Alghanem 2010 (Alghanem et al., 2010)	Yes				Yes				
Areeruk 2016 (Areeruk et al., 2016)			Yes	Yes	Yes	Yes		Yes	
Badawy 2015 (Badawy & Sakka, 2015)	Yes		Yes		Yes		Yes	Yes	
Bataille 2016 (Bataille et al., 2016)	Yes	Yes			Yes	Yes			
Batistaki 2019 (Batistaki et al., 2019)	Yes		Yes		Yes				
Benevides 2013 (Benevides et al., 2013)								Yes	Yes
Bianchin 2007 (Bianchin et al., 2007)	Yes		Yes		Yes				
Bilgin 2010 (Bilgin et al., 2010)			Yes						
Bisgaard 2003 (Bisgaard et al., 2003)								Yes	Yes
Coloma 2002 (Coloma et al., 2002)							Yes	Yes	Yes
Corcoran 2017 (Corcoran et al., 2017)	Yes	Yes							
De Oliveira 2011 (De Oliveira Jr. et al., 2011b)	Yes						Yes	Yes	
Elhakim 2002 (Elhakim et al., 2002)					Yes	Yes	Yes	Yes	
Feo 2006 (Feo et al., 2006)	Yes		Yes		Yes				
Fukami 2009 (Fukami et al., 2009)	Yes		Yes		Yes				
Gautam 2008 (Gautam et al., 2008)	Yes		Yes		Yes		Yes	Yes	
Hammas 2002 (Hammas et al., 2002)								Yes	
Ionescu 2014 (Ionescu et al., 2014)								Yes	
Jo 2012 (Jo et al., 2012)	Yes		Yes						
Jokela 2009 (Jokela et al., 2009)	Yes	Yes	Yes	Yes	Yes	Yes	Yes	Yes	
Kasagi 2013 (Kasagi et al., 2013)	Yes		Yes						
Kassim 2018 (Kassim et al., 2018)							Yes	Yes	
Ko-iam 2015 (Ko-Iam et al., 2015)			Yes					Yes	
Kurz 2015 (Kurz et al., 2015)	Yes		Yes						
Lee 2017 (Lee et al., 2017)		Yes		Yes		Yes	Yes	Yes	
Lim 2011 (Lim et al., 2011)	Yes		Yes		Yes			Yes	
Liu 1998 (Liu et al., 1998)								Yes	
Liu 1999 (Liu et al., 1999)					Yes		Yes	Yes	
Lopez-Olaondo 1996 (Lopez-Olaondo et al., 1996)								Yes	
Maddali 2003 (Maddali et al., 2003)			Yes						
Mathiesen 2009 (Mathiesen et al., 2009)	Yes	Yes			Yes	Yes		Yes	
McKenzie 1997 (McKenzie et al., 1997)	Yes				Yes			Yes	
Murphy 2011 (Murphy et al., 2011)	Yes	Yes	Yes	Yes				Yes	Yes
Murphy 2014 (Murphy et al., 2014)	Yes	Yes						Yes	Yes
Nesek-Adam 2007 (Nesek-Adam et al., 2007)	Yes	Yes	Yes	Yes			Yes		
Olajumoke 2013 (Olajumoke et al., 2013)									Yes
Pan 2008 (Pan et al., 2008)	Yes		Yes		Yes			Yes	Yes
Pauls 2015 (Pauls et al., 2015)					Yes			Yes	
Regasa 2020 (Regasa et al., 2020)								Yes	
Rothenberg 1998 (Rothenberg et al., 1998)			Yes					Yes	Yes
Ryu 2013 (Ryu et al., 2013)	Yes		Yes		Yes				
Sanchez-Ledesma 2002 (Sanchez-Ledesma et al., 2002)	Yes		Yes	Yes	Yes	Yes		Yes	
Sanchez-Rodriquez 2010 (Sanchez-Rodriguez et al., 2010)	Yes		Yes		Yes				
Shrestha 2014 (Shrestha et al., 2014)					Yes				
Sistla 2009 (Sistla et al., 2009)	Yes	Yes	Yes	Yes	Yes	Yes		Yes	
Thangaswamy 2010 (Thangaswamy et al., 2010)	Yes	Yes	Yes	Yes	Yes	Yes	Yes	Yes	
Tolver 2012 (Tolver et al., 2012)			Yes	Yes	Yes	Yes		Yes	
Viriyaroj 2008 (Viriyaroj et al., 2015)	Yes		Yes		Yes			Yes	
Wang 1999 (Wang et al., 1999)	Yes							Yes	
Wang 2000 (Wang et al., 2000)	Yes							Yes	
Wu 2009 (Wu et al., 2009)	Yes								
Yuksek 2003 (Yuksek et al., 2003)			Yes				Yes		

PACU LOS, post-anaesthesia care unit length of stay

Bias assessment judged seven studies to be low risk (Sanchez-Ledesma et al., 2002; Gautam et al., 2008; Kassim et al., 2018; McKenzie et al., 1997; Murphy et al., 2011; Thangaswamy et al., 2010; Tolver et al., 2012), 20 studies to have some concerns (Bataille et al., 2016; Jo et al., 2012; Alghanem et al., 2010; Badawy & Sakka, 2015; Benevides et al., 2013; Coloma et al., 2002; Feo et al., 2006; Fukami et al., 2009; Kasagi et al., 2013; Lee et al., 2017; Lim et al., 2011; Lopez-Olaondo et al., 1996; Murphy et al., 2014; Nesek-Adam et al., 2007; Olajumoke et al., 2013; Pan et al., 2008; Rothenberg et al., 1998; Sanchez-Rodriguez et al., 2010; Shrestha et al., 2014; Viriyaroj et al., 2015) and 25 to be high risk (Ko-Iam et al., 2015; Areeruk et al., 2016; Batistaki et al., 2019; Bianchin et al., 2007; Bilgin et al., 2010; Bisgaard et al., 2003; Corcoran et al., 2017; De Oliveira Jr. et al., 2011b; Elhakim et al., 2002; Hammas et al., 2002; Ionescu et al., 2014; Jokela et al., 2009; Kurz et al., 2015; Liu et al., 1998; Liu et al., 1999; Maddali et al., 2003; Mathiesen et al., 2009; Pauls et al., 2015; Regasa et al., 2020; Ryu et al., 2013; Sistla et al., 2009; Wang et al., 1999; Wang et al., 2000; Wu et al., 2009; Yuksek et al., 2003). For risk of bias (ROB) assessment, see Supplementary Table 2, Additional File 4 and Supplementary Fig. 1 and 2, Additional File 5.

### Pain scores

**Fig. 2:**
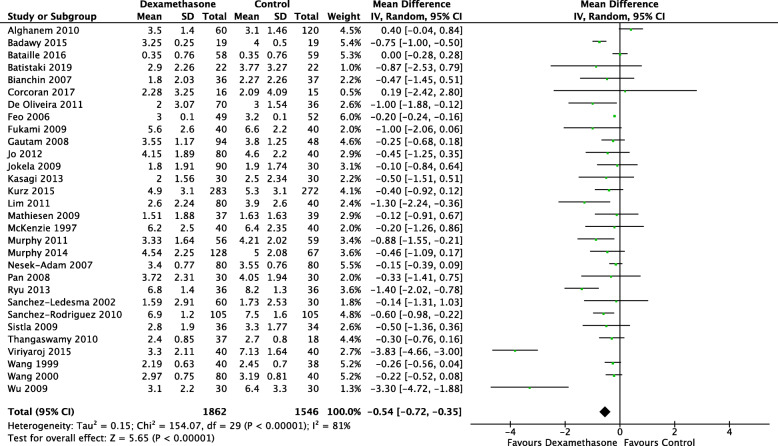
Forest plot for early (≤ 4 h) VAS pain scores at rest

Early pain scores at rest were recorded in 30 studies (3408 patients) (Bataille et al., 2016; Jo et al., 2012; Sanchez-Ledesma et al., 2002; Alghanem et al., 2010; Badawy & Sakka, 2015; Batistaki et al., 2019; Bianchin et al., 2007; Corcoran et al., 2017; De Oliveira Jr. et al., 2011b; Feo et al., 2006; Fukami et al., 2009; Gautam et al., 2008; Jokela et al., 2009; Kasagi et al., 2013; Kurz et al., 2015; Lim et al., 2011; Mathiesen et al., 2009; McKenzie et al., 1997; Murphy et al., 2011; Murphy et al., 2014; Nesek-Adam et al., 2007; Pan et al., 2008; Ryu et al., 2013; Sanchez-Rodriguez et al., 2010; Sistla et al., 2009; Thangaswamy et al., 2010; Viriyaroj et al., 2015; Wang et al., 1999; Wang et al., 2000; Wu et al., 2009) with a statistically significant reduction in pain in patients receiving dexamethasone (MD − 0.54; CI − 0.72, − 0.35; *I*^2^ 81%; *n* = 3408) (Fig. 2). The direction of result remained unchanged when the analysis was restricted to studies with pain (MD − 0.8; CI − 1.22, − 0.38; I^2^ 91%; *n* = 950) and non-pain (MD − 0.4; CI − 0.62, − 0.19; *I*^2^ 63%; *n* = 2458) primary outcomes.

Ten studies (1319 patients) reported early pain scores on movement (Bataille et al., 2016; Corcoran et al., 2017; Jokela et al., 2009; Lee et al., 2017; Mathiesen et al., 2009; Murphy et al., 2011; Murphy et al., 2014; Nesek-Adam et al., 2007; Sistla et al., 2009; Thangaswamy et al., 2010) with a statistically significant reduction in pain in patients who received dexamethasone (MD − 0.42; CI − 0.62, − 0.22; *I*^2^ 35%; *n* = 1319). The result trend did not vary when the analysis was limited to studies with non-pain (MD − 0.47; CI − 0.84, − 0.10; *I*^2^ 52%; *n* = 618) or pain (MD − 0.43; CI − 0.68, − 0.18; *I*^2^ 17%; *n* = 701) as the primary outcome.

Intermediate pain scores at rest were recorded in 27 studies (3022 patients) (Jo et al., 2012; Sanchez-Ledesma et al., 2002; Ko-Iam et al., 2015; Areeruk et al., 2016; Badawy & Sakka, 2015; Batistaki et al., 2019; Bianchin et al., 2007; Bilgin et al., 2010; Feo et al., 2006; Fukami et al., 2009; Gautam et al., 2008; Jokela et al., 2009; Kasagi et al., 2013; Kurz et al., 2015; Lim et al., 2011; Maddali et al., 2003; Murphy et al., 2011; Nesek-Adam et al., 2007; Pan et al., 2008; Rothenberg et al., 1998; Ryu et al., 2013; Sanchez-Rodriguez et al., 2010; Sistla et al., 2009; Thangaswamy et al., 2010; Tolver et al., 2012; Viriyaroj et al., 2015; Yuksek et al., 2003) and on movement in nine studies (1112 patients) (Sanchez-Ledesma et al., 2002; Areeruk et al., 2016; Jokela et al., 2009; Lee et al., 2017; Murphy et al., 2011; Nesek-Adam et al., 2007; Sistla et al., 2009; Thangaswamy et al., 2010; Tolver et al., 2012). There was a statistically significant reduction in intermediate pain scores both at rest (MD − 0.31; CI − 0.47, − 0.14; *I*^2^ 96%; *n* = 3022) and on movement (MD − 0.26; CI − 0.39, − 0.13; *I*^2^ 29%; *n* = 1112) in patients receiving dexamethasone. When analysis of intermediate pain scores at rest was restricted to studies with pain as the primary outcome, the direction of result remained (MD − 0.57; CI − 0.92, − 0.22; *I*^2^ 89%; *n* = 996); however, lost statistical significance when restricted to non-pain primary outcomes (MD − 0.18; CI − 0.39, 0.03; *I*^2^ 97%; *n* = 2026). Restricting the results for intermediate pain scores on movement to pain (MD − 0.33; CI − 0.45, − 0.21; *I*^2^ 0%; *n* = 747) and non-pain (MD − 0.16; CI − 0.25, − 0.07; *I*^2^ 0%; *n* = 365) primary outcomes did not change the direction of the result.

Late pain scores at rest were recorded in 25 studies (2443 patients) (Bataille et al., 2016; Sanchez-Ledesma et al., 2002; Alghanem et al., 2010; Areeruk et al., 2016; Badawy & Sakka, 2015; Batistaki et al., 2019; Bianchin et al., 2007; Elhakim et al., 2002; Feo et al., 2006; Fukami et al., 2009; Gautam et al., 2008; Jokela et al., 2009; Lim et al., 2011; Liu et al., 1999; Mathiesen et al., 2009; McKenzie et al., 1997; Pan et al., 2008; Pauls et al., 2015; Ryu et al., 2013; Sanchez-Rodriguez et al., 2010; Shrestha et al., 2014; Sistla et al., 2009; Thangaswamy et al., 2010; Tolver et al., 2012; Viriyaroj et al., 2015). There was a statistically significant reduction in pain scores in patients who received dexamethasone (MD − 0.38; CI − 0.52, − 0.24; *I*^2^ 88%; *n* = 2443). The direction of the result was unchanged when the study outcome was restricted to pain (MD − 0.42; CI − 0.68, − 0.16; *I*^2^ 90%; *n* = 1192) and non-pain (MD − 0.34; CI − 0.57, − 0.11; *I*^2^ 77%; *n* = 1251) primary outcomes.

Ten studies (1210 patients) (Bataille et al., 2016; Sanchez-Ledesma et al., 2002; Areeruk et al., 2016; Elhakim et al., 2002; Jokela et al., 2009; Lee et al., 2017; Mathiesen et al., 2009; Sistla et al., 2009; Thangaswamy et al., 2010; Tolver et al., 2012) reported late pain on movement with a statistically significant reduction in pain scores in patients who received dexamethasone (MD − 0.38; CI − 0.65, − 0.11; *I*^2^ 71%; *n* = 1210). Confining the results to non-pain primary outcomes did not change the result trend (MD − 0.49; CI − 0.95, − 0.03; *I*^2^ 59%; *n* = 387) but limiting to studies with pain as the primary outcome demonstrated no statistical significance (MD − 0.3; CI − 0.61, 0.00; *I*^2^ 66%; *n* = 823).

### Analgesic requirements

**Fig. 3:**
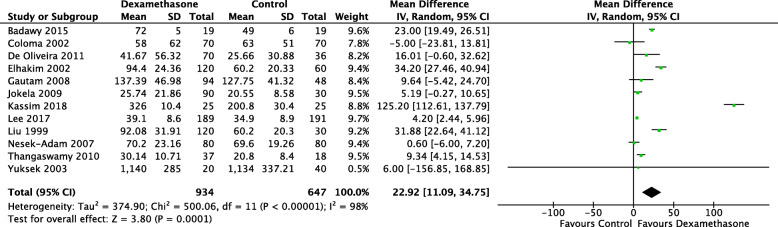
Forest plot for time to first analgesia in minutes

Time to first analgesia was recorded in 12 studies (1581 patients) (De Oliveira et al., 2011; Kassim et al., 2018; Lee et al., 2017; Elhakim et al., 2002; Jokela et al., 2009; Liu et al., 1999; Thangaswamy et al., 2010; Coloma et al., 2002; Yuksek et al., 2003; Gautam et al., 2008; Nesek-Adam et al., 2007; Badawy & Sakka, 2015) Listed as numbers and not the same as above. There was a statistically significant increase in time to first analgesia (minutes) in patients who received dexamethasone (MD 22.92.; CI 11.09, 34.75; *I*^2^ 99%; *n* = 1581) (Fig. 3). Restricting the analysis to studies with pain (MD 31.97; CI 13.35, 50.60; *I*^2^ 99%; *n* = 643) and non-pain primary outcomes (MD 15.17; CI 0.33, 30.02; *I*^2^ 91%; *n* = 938) did not affect the trend.

**Fig. 4:**
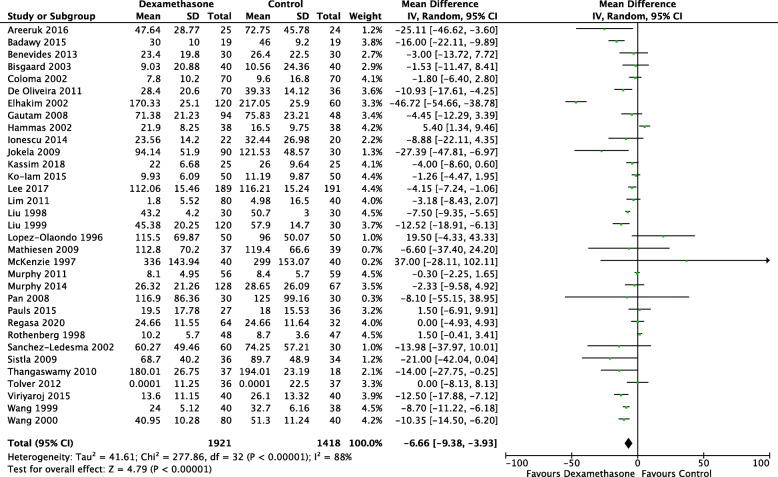
Forest plot for total postoperative opioid use in mg of oral morphine equivalents

Postoperative opioids were recorded in 33 studies (3339 patients) (Sanchez-Ledesma et al., 2002; Ko-Iam et al., 2015; Areeruk et al., 2016; Badawy & Sakka, 2015; Benevides et al., 2013; Bisgaard et al., 2003; Coloma et al., 2002; De Oliveira Jr. et al., 2011b; Elhakim et al., 2002; Gautam et al., 2008; Hammas et al., 2002; Ionescu et al., 2014; Jokela et al., 2009; Kassim et al., 2018; Lee et al., 2017; Lim et al., 2011; Liu et al., 1998; Liu et al., 1999; Lopez-Olaondo et al., 1996; Mathiesen et al., 2009; McKenzie et al., 1997; Murphy et al., 2011; Murphy et al., 2014; Pan et al., 2008; Pauls et al., 2015; Regasa et al., 2020; Rothenberg et al., 1998; Sistla et al., 2009; Thangaswamy et al., 2010; Tolver et al., 2012; Viriyaroj et al., 2015; Wang et al., 1999; Wang et al., 2000). However, there was variability in the type, administration and time of recorded opioids varying from one hour to five days postoperatively. There was a statistically significant reduction in opioid use (mg of oral morphine equivalents) in patients who received dexamethasone (MD − 6.66; CI − 9.38, − 3.93; *I*^2^ 88%; *n* = 3339) (Fig. 4). Statistical significance remained when the result was restricted to pain (MD − 8.35; CI − 11.64, − 5.07; *I*^2^ 58%; *n* = 1251) and non-pain (MD − 5.50; CI − 9.15, − 1.85; *I*^2^ 91%; *n* = 2088) primary outcomes. Visual inspection of the funnel plots for total opioid requirements and early pain scores at rest do not suggest evidence of significant reporting or publication bias (see Supplementary Fig. 3 and Fig. 4, Additional File 6).

### Time to PACU discharge

Nine studies (947 patients) reported time to discharge from PACU (Benevides et al., 2013; Bisgaard et al., 2003; Coloma et al., 2002; De Oliveira Jr. et al., 2011b; Murphy et al., 2011; Murphy et al., 2014; Olajumoke et al., 2013; Pan et al., 2008; Rothenberg et al., 1998). There was no difference in time to PACU discharge between patients who received dexamethasone and those who did not (MD − 3.82; CI − 10.87, 3.23; *I*^2^ 59%; *n* = 947). Removing the single study with pain as the primary outcome and restricting the analysis to non-pain (MD − 4.37; CI − 12.10, 3.37; *I*^2^ 54%; *n* = 867) had no impact on the result.

### Subgroup analyses

Subgroup analyses of general anaesthesia in combination with either central neuraxial blockade (GA + CNB) or regional anaesthesia (GA + RA) were previously documented (CRD42020176202) (Research NIfH, 2020). Patients received GA + CNB in three studies; spinal with intrathecal morphine (Sanchez-Ledesma et al., 2002), epidural administration of morphine and fentanyl (Yuksek et al., 2003) and a small proportion of both the intervention and control groups received an epidural in one study (Kurz et al., 2015). The subset of study data was not available in this study (Kurz et al., 2015). One study documented the use of regional anaesthesia with either transversus abdominal plane block or rectus sheath block (Regasa et al., 2020). Given the limited data, these predefined subgroup analyses were not undertaken.

The planned dosing subgroup analyses were undertaken for a single but not multiple doses of dexamethasone. Doses were grouped pragmatically into three categories to correspond with clinical practice; low dose 1.25–5 mg, intermediate dose 6.4–10 mg and high dose 11–20 mg. For early pain scores at rest, both low (MD − 0.55; CI − 1.04, − 0.07; *I*^2^ 66%; *n* = 1023) and intermediate (MD − 0.55; CI − 0.76, − 0.34; *I*^2^ 83%; *n* = 2265) demonstrated benefit with no impact from high dose (MD − 0.21; CI − 1.02, 0.60; *I*^2^ 0%; *n* = 120). For early pain scores on movement, only intermediate dose (MD − 0.48; CI − 0.75, − 0.21; *I*^2^ 47; *n* = 587) demonstrated benefit with no impact from low (MD − 0.34; CI − 0.67, 0.00; *I*^2^ 0%; *n* = 692) or high dose (MD − 0.40; CI − 1.79, 0.99; *n* = 40).

For 4–24-h pain scores, again, there was evidence of dose response for intermediate dose at rest (MD − 0.36; CI − 0.53, − 0.18; *I*^2^ 96%; *n* = 2221) and on movement (MD − 0.25; CI − 0.37, − 0.13; *I*^2^ 22%, *n* = 1005). There was a lack of statistical significance for low (MD 0.22; CI − 0.15, 0.58; *I*^2^ 0%, *n* = 666) and high dose (MD − 0.05; CI − 0.76, 0.66; *I*^2^ 0%; *n* = 135) at rest and low (MD − 0.12; CI − 0.77, 0.54; *I*^2^ 0%; *n* = 67) and high dose (MD − 0.70; CI − 2.62, 1.22; *n* = 40) on movement.

**Fig. 5:**
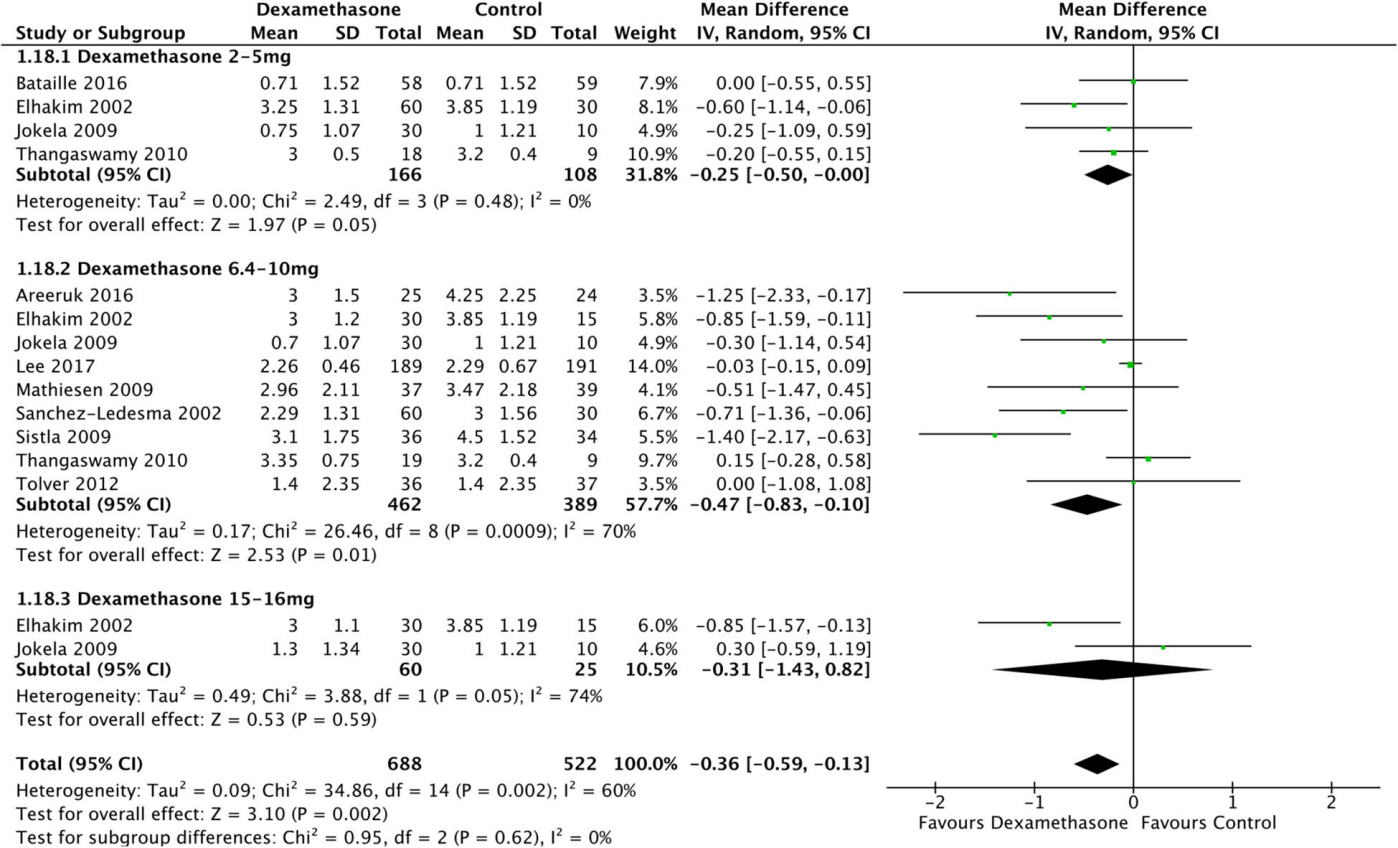
Forest plot for dexamethasone dosing late (≥ 24 h) VAS pain scores at rest

Intermediate dose remained statistically significant (MD − 0.42; CI − 0.62, − 0.22; *I*^2^ 84%; *n* = 1847) for late pain scores at rest but low (MD − 0.08; CI − 0.22, 0.06; *I*^2^ 19%; *n* = 431) and high (MD − 0.51; CI − 1.32, 0.30; *I*^2^ 66%; *n* = 165) dose dexamethasone demonstrated no difference (Fig. 5). This pattern was mirrored in late pain scores on movement; low (MD − 0.25; CI − 0.5, 0.00; *I*^2^ 0%; *n* = 274), intermediate (MD − 0.47; CI − 0.83, − 0.10; *I*^2^ 70%; *n* = 851) and high dose (MD − 0.31; CI − 1.43, 0.82; *I*^2^ 74%; *n* = 85).

Time to first analgesia was increased with intermediate dose (MD 27.76; CI 13.96, 41.55; *I*^2^ 98%; *n* = 1034) but low (MD 11.58; CI − 0.34, 23.5; *I*^2^ 89%; *n* = 462) and high dose had no impact (MD 25.44; CI − 2.23, 53.12; *I*^2^ 86%; *n* = 85). Again, a statistically significant reduction in postoperative opioid requirements was maintained for intermediate dose (MD − 7.20; CI − 9.77, − 4.64; *I*^2^ 80%; *n* = 2402) but low (MD − 8.14; CI − 16.72, 0.44; *I*^2^ 89%; *n* = 677) and high dose dexamethasone (MD − 19.26; CI − 57.79, 19.28; *I*^2^ 94%; *n* = 260) demonstrated no difference.

Subgroup analysis did not impact time to PACU discharge with no difference from low (MD 0.27; CI − 6.72, 7.27; *I*^2^ 40%; *n* = 385), intermediate (MD − 9.56; CI − 24.56, 5.44; *I*^2^ 65%; *n* = 467) or high (MD − 3.76; CI − 15.77, 8.25; *n* = 95) dose dexamethasone (see supporting information, Appendix 3).

Timing of administration subgroup analyses of dexamethasone were also performed. This was categorised as preoperative (before anaesthetic induction), intraoperative (anaesthetic induction and to extubation) and postoperative (after extubation). The timing subgroup analyses demonstrated a global reduction in pain scores from preoperative administration of dexamethasone for all pain scores both at rest and on movement. In contrast, intraoperative administration only reduced late pain scores at rest.

Preoperative dexamethasone significantly increased time to first analgesia (MD 28.13; CI 14.57, 41.68; *I*^2^ 98%; *n* = 1281), but there was no difference from intraoperative administration (MD − 0.01; CI − 6.24, 6.21; *I*^2^ 0%; *n* = 300). Additionally, preoperative dexamethasone decreased total opioid administration (MD − 8.55; CI − 12.34, − 4.76; *I*^2^ 89%; *n* = 2214) with no effect from intraoperative administration (MD − 2.18; CI − 5.93, 1.56; *I*^2^ 83%; *n* = 1065). Postoperative dexamethasone (MD − 12.00; CI − 17.45, − 6.55; *n* = 40) decreased opioid administration but this was based on results from a single study (Wang et al., 2000). Time to PACU discharge remained unaffected by dexamethasone timing; preoperative (MD − 12.55; CI − 30.73, 5.63; *I*^2^ 62%; *n* = 361), intraoperative (MD − 0.56; CI − 7.41, 6.29; *I*^2^ 57%; *n* = 586) (For additional forest plots see Additional File 7).

## Conclusions

To our knowledge, this is the largest systematic review and meta-analysis investigating the effect of perioperative dexamethasone on postoperative pain in adults undergoing elective abdominal surgery under general anaesthesia and the first to demonstrate an important analgesic effect in this surgical cohort.

Our analyses demonstrated a statistically significant reduction in early, intermediate and late pain scores both at rest and on movement. Sub-group analyses revealed that intermediate dose (6.4–10 mg) effectively decreased pain at all time intervals both at rest and on movement. However, low dose (1.25–5 mg) only affected early pain scores at rest while high dose (11–20 mg) had no impact on any pain scores. Preoperative administration of dexamethasone demonstrated a global reduction on all pain scores. Intraoperative administration was more beneficial in reducing late pain scores at rest but failed to impact pain at any other time period. Dexamethasone also reduced the total postoperative opioid requirements and increased the time to first analgesia with intermediate dose (6.4–10 mg) and preoperative administration demonstrating the greatest impact. Time to PACU discharge was not altered by dexamethasone at any dose or time and is likely to be influenced by external factors (Samad et al., 2006). However, this is contrary to previous findings which have questionable clinical significance (Waldron et al., 2013).

Dexamethasone’s established anti-inflammatory properties have ensured it is a widely used effective perioperative anti-emetic (Moore, 2018; Holte & Kehlet, 2002; De Oliveira Jr. et al., 2013). In abdominal surgery, glucocorticoids reduce pro-inflammatory mediators and phospholipase required for pain pathways allowing its analgesic benefits to be increasingly recognized (Moore, 2018; De Oliveira Jr. et al., 2011a; Waldron et al., 2013; Holte & Kehlet, 2002). Enhanced recovery pathways encouraging earlier mobility have boosted the demand for opioid-sparing multimodal analgesia in patients undergoing abdominal surgery (Gustafsson et al., 2019; Nelson et al., 2019; Nygren et al., 2012; Thorell et al., 2016). Dexamethasone has, therefore, an important role in postoperative analgesia with additional benefit for multimodal analgesic regimes in this patient population. However, full analgesic effect is unlikely from the commonly used lower anti-emetic dose and intermediate dose (6.4–10 mg) is necessary to produce global reductions in pain scores, increase time to first analgesia and reduce opioid requirements (Moore, 2018; De Oliveira Jr. et al., 2013). Additionally, timing of administration is crucial as the analgesic benefits of preoperative dexamethasone far outweigh administration at induction as recommended for antiemetic effect (Gan et al., 2020).

One of the major strengths of this review is inclusion of a large number of studies and participants of a relatively homogenous surgical population. This allows the results to inform future clinical practice and guidelines in moderate and major abdominal surgery. A previous systematic review failed to demonstrate a reduction in early pain scores on movement from dexamethasone administration, likely due to small numbers (Waldron et al., 2013). This new finding is potentially significant for enhanced recovery regimes where early movement after abdominal surgery is encouraged (Gustafsson et al., 2019; Nelson et al., 2019; Nygren et al., 2012; Thorell et al., 2016). In addition, investigation of dexamethasone’s effect on intermediate pain scores is novel and provides further evidence of its analgesic effects (De Oliveira Jr. et al., 2011a; Waldron et al., 2013). Through subgroup analyses, we have provided clarification on the debated perioperative dosing and given strength to the previously suggested preoperative timing (De Oliveira Jr. et al., 2011a; Waldron et al., 2013). Despite demonstrating a globally statistically significant reduction in postoperative pain scores, it is important to remember that the clinical significance of this is uncertain. The increase in time to first analgesia and reduction in postoperative opioids is likely to have more clinical impact on patients undergoing abdominal surgery. When studies with regional anaesthesia were removed, a statistically significant reduction in postoperative opioids (MD − 6.87; CI − 9.70, − 4.05; *I*^2^ 89%; *n* = 3153) and increased time to first analgesia (MD 23.01; CI 11.14, 34.88; *I*^2^ 98%; *n* = 1521) remained.

There are a number of limitations in our review. Firstly, results could potentially be biased by selective reporting and missing outcome data, but the funnel plots were reassuring (Chen et al., 2020; D'Souza et al., 2011; Kirdak et al., 2008; Bala et al., 2014; Bilgin et al., 2004; Chu et al., 2008; Karaman et al., 2013; McKenzie et al., 1994; Ramesh, 2011; Wang et al., 2002; Zargar-Shoshtari et al., 2009). Secondly, as the latest pain score was extracted from each time interval, there could be significant variation in the timing which may explain some of the statistical heterogenicity in intermediate and late pain scores. Late pain scores varied from 24 h to 4 days, with later pain scores less likely to demonstrate statistical significance potentially influencing the results. The variation in timing of recorded postoperative opioid consumption, from 1 h to 5 days, may also account for some of the statistical heterogenicity. Thirdly, results from the high dose and postoperative subgroup analyses should be interpreted with caution given the low numbers available. In addition, we did not investigate the impact of adverse effects of dexamethasone administration as this has previously been done (De Oliveira Jr. et al., 2011a; Waldron et al., 2013; Polderman et al., 2018). However, when reported, adverse features reported were similar between intervention and control groups and not attributed to dexamethasone administration.

Furthermore, pain was the primary outcome in less than half the studies but when analyses were restricted to studies with pain as the primary outcome all results remained statistically significant except late pain scores on movement. In addition, pain scores on movement were less likely to be reported potentially reducing the strength of the sensitivity analyses. Pain scores on movement should be the focus of future studies given the drive for postoperative mobilisation.

Additionally, due to lack of data, we were unable to perform our prespecified subgroup analyses GA + CNB and GA + RA. Dexamethasone may impact on postoperative pain in combination with general and regional anaesthesia, but it is unclear if this can be translated to the general surgical population (Chen et al., 2018; Fan et al., 2018; Pehora et al., 2017). It is our opinion that this question remains unanswered and should guide future research.

Unfortunately, nearly half of all studies were deemed high ROB, frequently due to selection of the reported result with failure to report all measured pain scores. As the majority of studies had a non-pain primary outcome, ROB assessment at study rather than outcome level would have impacted these results. ROB assessment highlighted issues with study methodology, with inadequate allocation concealment in nearly half of all studies, and trialists should be reminded of reporting guidelines for RCTs (Moher et al., 2010). Additionally, the type of analysis was infrequently documented, and we judged nearly half of all studies undertook a per-protocol analysis due to exclusions of protocol violations and post-randomisation participants for reasons not pre-specified. Some exclusions are justified in a modified intention-to-treat (mITT) analysis, but we exercised caution using this label due to ambiguity over the definition (Abraha & Montedori, 2010; Gupta, 2011). We feel clarification of mITT criteria is essential to avoid subjectivity of future ROB assessments. However, the completeness of outcome data provides some reassurance over the safety and lack of adverse features of dexamethasone.

In conclusion, a single perioperative dose of intravenous dexamethasone reduces early, intermediate and late pain scores both at rest and on movement, opioid requirements and increases time to first analgesia in patients undergoing elective abdominal surgery. Preoperative administration of intermediate dose is likely to have the greatest impact on outcomes.

## Supplementary Information

**Additional file 1.** Search Strategy

**Additional file 2.** List of included items on data extraction form and relevant dropdown list. Dropdown lists were used when possible to reduce the amount of free text.

**Additional file 3: Supplementary Table 1.** Table of morphine equivalents used to convert from intravenous or oral opioids to oral morphine

**Additional file 4: Supplementary Table 2.** Tabular Risk of Bias Assessment

**Additional file 5: Supplementary Figure 1 and Supplementary Figure 2.** Summary risk of bias chart and weighted risk of bias chart.

**Additional file 6: Supplementary Figure 3.** Funnel plot for total postoperative opioid requirements.

**Additional file 7.** Additional forest plots

## Data Availability

All data collected and analysed for the current study are available from the corresponding author on reasonable request.

## Abbreviations

CENTRAL: Cochrane Central Register of Controlled Trials
CI: Confidence interval
CINAHL: Cumulative Index to Nursing and Allied Health Literature
CNB: Central neuraxial blockade
EMBASE: Exerpta Medica Database
GA: General anaesthesia
ICTRP: International Clinical Trials Registry Portal
MEDLINE: Medical Analysis and Retrieval System Online
MD: Mean difference
mITT: Modified intention to treat
PACU: Post anaesthesia care unit
PRISMA: Preferred reporting items for systematic reviews and meta-analysis
RA: Regional anaesthesia
RevMan: Review manager
RCT: Randomised controlled trial
ROB: Risk of bias
PONV: Postoperative nausea and vomiting
WHO: World Health Organisation
