# Supplementary material for: What is the impact of dexamethasone on postoperative pain in adults undergoing general anaesthesia for elective abdominal surgery: a systematic review and meta-analysis
Source: Perioper Med (Lond). 2022 Mar 24;11:13. doi: 10.1186/s13741-022-00243-6 (PMC8942613; doi:10.1186/s13741-022-00243-6)
Supplement: Supplementary file 1 — Additional file 1. Search Strategy [file 13741_2022_243_MOESM1_ESM.docx]

**Additional File 1**

**Search Strategy**

**Medical Analysis and Retrieval System Online (MEDLINE)**

1. “Digestive System Surgical Procedures”[Mesh] OR “Colorectal Surgery”[Mesh] OR “Gynecologic surgical procedures”[Mesh] OR “Urologic Surgical Procedures, Male”[Mesh] OR “Urologic surgical procedures”[Mesh]
2. “Procedure, Digestive System Surgical” OR “Surgical Procedure, Digestive System” OR “Surgical Procedures, Digestive System” OR “Digestive System Surgical Procedure” OR “Procedures, Digestive System Surgical” OR “Gastrointestinal Surgical Procedure” OR “Gastrointestinal Surgical Procedures” OR “Surgical Procedure, Gastrointestinal” OR “Surgical Procedures, Gastrointestinal” OR “Procedure, Gastrointestinal Surgical” OR “Procedures, Gastrointestinal Surgical” OR “Surgery Specialty, Colon and Rectal” OR “Surgery, Colorectal” OR “Colon and Rectal Surgery Specialty” OR “Colon Surgery Specialty” OR “Specialty, Colon Surgery” OR “Surgery Specialty, Colon” OR “Proctology” OR “Specialty, Rectal Surgery” OR “Surgery Specialty, Rectal” OR “Rectal Surgery Specialty” OR “Procedures, Gynecologic Surgical” OR “Surgical Procedure, Gynecologic” OR “Surgery, Gynecological” OR “Gynecological Surgeries” OR “Gynecological Surgery” OR “Surgeries, Gynecological” OR “Gynecologic Surgical Procedure” OR “Surgical Procedures, Gynecologic” OR “Gynecological Surgical Procedure” OR “Gynecological Surgical Procedures” OR “Procedure, Gynecological Surgical” OR “Procedures, Gynecological Surgical” OR “Surgical Procedure, Gynecological” OR “Surgical Procedures, Gynecological” OR “Procedure, Gynecologic Surgical” OR “Gynecologic Surgery” OR “Gynecologic Surgeries” OR “Surgeries, Gynecologic” OR “Surgery, Gynecologic” OR “Surgery, Male Urologic” OR “Urologic Surgery, Male” OR “Male Urologic Surgical Procedure” OR “Male Urologic Surgical Procedures” OR “Procedure, Urologic Surgical, Male” OR “Procedures, Urologic Surgical, Male” OR “Surgical Procedure, Urologic, Male” OR “Surgical Procedures, Urologic, Male” OR “Urologic Surgical Procedure, Male” OR “Male Urological Surgical Procedure” OR “Male Urological Surgical Procedures” OR “Procedure, Urological Surgical, Male” OR “Procedures, Urological Surgical, Male” OR “Surgical Procedure, Urological, Male” OR “Surgical Procedures, Urological, Male” OR “Urological Surgical Procedure, Male” OR “Urological Surgical Procedures, Male” OR “Urological Surgery, Male” OR “Male Urological Surgery” OR “Surgery, Male Urological” OR “Male Urologic Surgery” OR “Procedures, Urologic Surgical” OR “Surgical Procedure, Urologic” OR “Urological Surgical Procedures” OR “Procedure, Urological Surgical” OR “Procedures, Urological Surgical” OR “Surgical Procedure, Urological” OR “Surgical Procedures, Urological” OR “Urological Surgical Procedure” OR “Urologic Surgical Procedure” OR “Procedure, Urologic Surgical” OR “Surgical Procedures, Urologic”
3. 1 or 2
4. “Glucocorticoids”[Mesh] OR “Glucocorticoids”[Pharmacological Action] OR “Dexamethasone”[Mesh] OR “Dexamethasone Isonicotinate”[Mesh] OR “Isonicotinic Acids”[Mesh] OR “Adrenal Cortex Hormones”[Mesh]
5. “Glucocorticoid” OR “Glucocorticoid Effect” OR “Effect, Glucocorticoid” OR “Glucorticoid Effects” OR “Effects, Glucorticoid” OR “Hexadecadrol” OR “Decameth” OR “Dexasone” OR “Dexpak” OR “Oradexon” OR “Decaject” OR “Decaject-L.A. ” OR “Decaject L.A. ” OR “Hexadrol” OR “Isonicotinate, Dexamethasone” OR “Acids, Isonicotinic” OR “Isonicotinic Acid” OR “Acid, Isonicotinic” OR “Hormones, Adrenal Cortex” OR “Corticosteroids” OR “Corticoids”
6. “Glucocort$” OR “Dexameth$” OR “Cortico$”
7. 4 or 5 or 6
8. “Pain”[Mesh] OR “Acute Pain”[Mesh] OR “Nocioceptive Pain”[Mesh] “Visceral Pain”[Mesh] OR “Pain Measurement”[Mesh] OR “Pain, Procedural”[Mesh] OR “Pain, Postoperative”[Mesh] OR “Abdominal Pain”[Mesh] OR “Nausea”[Mesh] OR “Postoperative Nausea and Vomiting”[Mesh] OR “Vomiting”[Mesh]
9. “Pain, Burning” OR “Burning Pain” OR “Burning Pains” OR “Pains, Burning” OR “Suffering, Physical” OR “Physical Suffering” OR “Physical Sufferings” OR “Sufferings, Physical” OR “Pain, Migratory” OR “Migratory Pain” OR “Migratory Pains” OR “Pains, Migratory” OR “Pain, Radiating” OR “Pains, Radiating” OR “Radiating Pain” OR “Radiating Pains” OR “Pain, Splitting” OR “Pains, Splitting” OR “Splitting Pain” OR “Splitting Pains” OR “Ache” OR “Aches” OR “Pain, Crushing” OR “Crushing Pain” OR “Crushing Pains” OR “Pains, Crushing” OR “Acute Pains” OR “Pain, Acute” OR “Pains, Acute” OR “Nociceptive Pains” OR “Pain, Nociceptive” OR “Pains, Nociceptive” OR “Pain, Visceral” OR “Pains, Visceral” OR “Visceral Pains” OR “Tissue Pain” OR “Pain, Tissue” OR “Pains, Tissue” OR “Tissue Pains” OR “Somatic Pain” OR “Pain, Somatic” OR “Pains, Somatic” OR “Somatic Pains” OR “Abdominal Pains” OR “Pain, Abdominal” OR “Pains, Abdominal” OR “Colicky Pain” OR “Colicky Pains” OR “Pain, Colicky” OR “Pains, Colicky” OR “Measurement, Pain” OR “Measurements, Pain” OR “Pain Measurements” OR “Assessment, Pain” OR “Assessments, Pain” OR “Pain Assessments” OR “Pain Assessment” OR “Analgesia Tests” OR “Analgesia Test” OR “Test, Analgesia” OR “Tests, Analgesia” OR “Nociception Tests” OR “Nociception Test” OR “Test, Nociception” OR “Tests, Nociception” OR “McGill Pain Questionnaire” OR “Pain Questionnaire, McGill” OR “Questionnaire, McGill Pain” OR “McGill Pain Scale” OR “Pain Scale, McGill” OR “Scale, McGill Pain” OR “Visual Analog Pain Scale” OR “Visual Analogue Pain Scale” OR “Analogue Pain Scale” OR “Analogue Pain Scales” OR “Pain Scale, Analogue” OR “Pain Scales, Analogue” OR “Scale, Analogue Pain” OR “Scales, Analogue Pain” OR “Analog Pain Scale” OR “Analog Pain Scales” OR “Pain Scale, Analog” OR “Pain Scales, Analog” OR “Scale, Analog Pain” OR “Scales, Analog Pain” OR “Formalin Test” OR “Formalin Tests” OR “Test, Formalin” OR “Tests, Formalin” OR OR “Procedural Pain” OR “Postoperative Pain” OR “Postoperative Pains” OR “PONV” OR “ Nausea and Vomiting, Postoperative” OR “Vomiting, Postoperative” OR “Postoperative Emesis” OR “Postoperative Vomiting” OR “Emesis, Postoperative” OR “Emeses, Postoperative” OR “Postoperative Emeses” OR “Postoperative Nausea” OR “Nausea, Postoperative” OR “Emesis”

# 8 or 9

# randomized controlled trial [pt]

# controlled clinical trial [pt]

# randomized [tiab]

# placebo [tiab]

# drug therapy [sh]

# randomly [tiab]

# trial [tiab]

# groups [tiab]

# 11 or 12 or 13 or 14 or 15 or 16 or 17 or 18

# humans [mh]

# 19 and 20

# 3 and 7 and 10 and 21

**Exerpta Medica Database (EMBASE)**

1. exp colorectal surgery/ or exp gynecologic surgery/
2. abdominal surgery/ or abdominal wall closure/ or biliary tract surgery/ or gastrointestinal surgery/ or hernioplasty/ or herniorrhaphy/ or herniotomy/ or laparoscopy/ or laparotomy/ or liver surgery/ or omentectomy/ or omentoplasty/ or peritoneum lavage/ or spleen surgery/ or urologic surgery/ or gynecologic surgery/ or male genital system surgery/ or sterilization reversal/ or urinary tract surgery/
3. (“colon and rectal surgery, special*” or “coloproctotomy” or “proctocolonic surgery” or “surgery, colorectal” or “intestine surger*” or “colorectal anastomosis” or “hartmann procedure” or “proctocolectomy” or “abdomen surger*” or “abdominal operation” or “digestive system surgical procedure*” or “surgery, abdominal” or “upper abdomen surgery” or “ pelvis surgery”).mp. [mp=title, abstract, heading word, drug trade name, original title, device manufacturer, drug manufacturer, device trade name, keyword, floating subheading word, candidate term word]
4. (“gyn?ecolog* surgery” or “gyn?ecolog* operation” or “gyn?ecolog* surgical procedure*” or “operative gyn?ecolog*” or “uro* surgery” or “uro* operation” or “uro* surgical procedure*” or “urogen* surgery” or “urogen* tract surgery” or “genitour* surgery” or “urogen* surg* procedure*”).mp. [mp=title, abstract, heading word, drug trade name, original title, device manufacturer, drug manufacturer, device trade name, keyword, floating subheading word, candidate term word]
5. 1 or 2 or 3 or 4
6. exp dexamethasone/ or exp dexamethasone isonicotinate/ or exp glucocorticoid/ or exp corticosteroid/
7. (dexamethasone or “dexamethasone isonicotinate” or dexamethason* or “glucocorticoid drug” or “glucocorticoid hormone” or “glucocorticoid steroid” or glucocorticoids or “glucocorticoids, synthetic” or glucocorticoidsteroid or glucocorticosteroid or glucocorticoid or glycocorticoid or glycocorticosteroid or glucocorticoid* or glucosteroid* or glucocorticosteroid* or “adrenal cortex hormone” or “adrenal cortex hormones” or “adrenal cortical hormone” or “ adrenal cortical hormones” or “adrenal cortical steroid” or “adrenal steroid” or “adrenal steroid hormone” or “adreno cortical steroid” or “adreno corticosteroid” or “adrenocortical hormone” or “adrenocorticosteroid” or “cortical steroid” or “cortico steroid” or corticoid or corticoid* or “corticosteroid agent” or “corticosteroid calcium” or “corticosteroid hormone” or corticosteroids or “corticosteroids, systemic” or corticosteroid* or “steroid hormone”).mp. [mp=title, abstract, heading word, drug trade name, original title, device manufacturer, drug manufacturer, device trade name, keyword, floating subheading word, candidate term word]
8. 6 or 7
9. exp pain/ or exp pain assessment/ or exp pain measurement/ or exp postoperative pain/ or exp nocioceptive pain/ or exp postoperative nausea/ or exp postoperative nausea/ or exp “postoperative nausea and vomiting”/ or exp postoperative vomiting/ or exp vomiting/ or exp “nausea and vomiting”/
10. (“acute pain” or “deep pain” or “lightning pain” or “pain response” or “treatment related pain” or “pain scale” or “disease assessment” or “Faces Pain Scale” or “McGill Pain Questionnaire” or “Memorial Pain Assessment Card” or “visual analogue scale” or pain or “algormetry” or “numeric rating scale” or “pain, postoperative” or “post operation pain” or “nausea, postoperative” or nausea or “postoperative nausea and vomiting” or PONV or “nausea and vomiting” or “postoperative nausea” or “postoperative vomiting” or “postoperative emesis” or “vomiting, postoperative” or vomiting or emesia or emesis or vomition or vomitus).mp. [mp=title, abstract, heading word, drug trade name, original title, device manufacturer, drug manufacturer, device trade name, keyword, floating subheading word, candidate term word]
11. 9 or 10
12. (random* or factorial* or crossover* or cross over* or cross-over* or placebo* or doubl* blind* or singl* blind* or assign* or allocat* or volunteer*).af.
13. exp crossover-procedure/ or exp double blind procedure/ or exp randomized controlled trial/ or exp single blind procedure/
14. 12 or 13
15. 5 and 8 and 11 and 14

**Cochrane Central Register of Controlled Trials (CENTRAL) in the Cochrane library**

1. MeSH descriptor: ([Digestive System Surgical Procedures] or [Colorectal Surgery] or [Gynecologic surgical procedures] or [Urologic Surgical Procedures, Male]) explode all trees
2. (Surgery Specialty, Colon and Rectal or Colon and Rectal Surgery Specialty or Surgery Specialty, Colon or Specialty, Colon Surgery or Colon Surgery Specialty or Rectal Surgery Specialty or Specialty, Rectal Surgery or Surgery Specialty, Rectal or Surgery, Colorectal or Proctology or Procedures, Digestive System Surgical or Surgical Procedure, Digestive System or Digestive System Surgical Procedure or Procedure, Digestive System Surgical or Surgical Procedures, Digestive System or Surgical Procedures, Gastrointestinal or Surgical Procedure, Gastrointestinal or Procedures, Gastrointestinal Surgical or Gastrointestinal Surgical Procedure or Gastrointestinal Surgical Procedures or Procedure, Gastrointestinal Surgical or Procedures, Gynecologic Surgical or Procedures, Gynecological Surgical or Gynecological Surgical Procedure or Surgeries, Gynecological or Gynecologic Surgery or Surgical Procedure, Gynecologic or Gynecological Surgeries or Surgical Procedures, Gynecologic or Gynecologic Surgeries or Surgical Procedures, Gynecological or Surgeries, Gynecologic or Gynecological Surgery; Surgery, Gynecologic or Gynecological Surgical Procedures or Gynecologic Surgical Procedure or Procedure, Gynecological Surgical or Procedure, Gynecologic Surgical or Surgical Procedure, Gynecological or Surgery, Gynecological or Procedures, Digestive System Surgical or Surgical Procedure, Digestive System or Digestive System Surgical Procedure or Procedure, Digestive System Surgical or Surgical Procedures, Digestive System or Surgical Procedures, Gastrointestinal or Surgical Procedure, Gastrointestinal or Procedures, Gastrointestinal Surgical or Gastrointestinal Surgical Procedure or Gastrointestinal Surgical Procedures or Procedure, Gastrointestinal Surgical)
3. 1 or 2
4. MeSH descriptor: ([Glucocorticoids] or [Dexamethasone] or [Dexamethasone Isonicotinate] or [Isonicotinic Acids] or [Adrenal Cortex Hormones]
5. (Glucocorticoid Effect or Effect, Glucocorticoid or Glucorticoid Effects or Effects, Glucorticoid or Glucocorticoid or Hexadecadrol or Dexasone or Dexpak or Decameth or Decaject or Oradexon or Hexadrol or Decaject-L.A. or Decaject L.A. or Isonicotinate, Dexamethasone or Acids, Isonicotinic or Isonicotinic Acid or Acid, Isonicotinic or Hormones, Adrenal Cortex or Corticosteroids or Corticoids)
6. 4 or 5
7. MeSH descriptor: ([Pain] or [Acute Pain] or [Pain, Procedural] or [Pain, Postoperative] or [Nocioceptive Pain] or [Visceral Pain] or [Abdominal Pain] or [Pain Measurement] or [Postoperative Nausea and Vomiting])
8. (Pains, Acute or Acute Pains or Pain, Acute or Pain, Abdominal or Pains, Abdominal or Abdominal Pains or Pain, Colicky or Colicky Pain or Colicky Pains or Pains, Colicky or Procedural Pain or Postoperative Pain or Postoperative Pains or Tissue Pain or Pain, Tissue or Tissue Pains or Pains, Tissue or Somatic Pains or Pain, Somatic or Somatic Pain or Pains, Somatic or Pain, Nociceptive or Pains, Nociceptive or Nociceptive Pains or Pain, Burning or Burning Pains or Pains, Burning or Burning Pain or Pain, Crushing or Crushing Pains or Pains, Crushing or Crushing Pain or Migratory Pain or Migratory Pains or Pains, Migratory or Pain, Migratory or Pain, Radiating or Radiating Pain or Pains, Radiating or Radiating Pains or Pain, Splitting or Splitting Pains or Splitting Pain or Pains, Splitting or Pains, Visceral or Visceral Pains or Pain, Visceral or Formalin Tests or Test, Formalin or Formalin Test or Tests, Formalin or Visual Analog Pain Scale or Visual Analogue Pain Scale or Scales, Analogue Pain or Pain Scale, Analog or Pain Scales, Analogue or Analogue Pain Scale or Analog Pain Scale or Analogue Pain Scales or Scale, Analog Pain or Analog Pain Scales or Pain Scale, Analogue or Scales, Analog Pain or Scale, Analogue Pain or Pain Scales, Analog or Pain Questionnaire, McGill or McGill Pain Scale or McGill Pain Questionnaire or Scale, McGill Pain or Questionnaire, McGill Pain or Pain Scale, McGill or Pain Assessments or Assessment, Pain or Test, Analgesia or Nociception Test or Tests, Analgesia or Analgesia Tests or Pain Measurements or Analgesia Test or Pain Assessment or Measurements, Pain or Assessments, Pain or Tests, Nociception or Measurement, Pain or Test, Nociception or Nociception Tests or PONV or Nausea and Vomiting, Postoperative or Postoperative Vomiting or Vomiting, Postoperative or Postoperative Emeses or Emeses, Postoperative or Emesis, Postoperative or Postoperative Emesis or Nausea, Postoperative or Postoperative Nausea or Vomiting or Nausea)
9. 7 or 8
10. 3 and 6 and 9

**Cumulative Index to Nursing and Allied Health Literature (CINAL)**

1. (MH "Biliary Tract Surgical Procedures+") OR (MH "Surgery, Digestive System+") OR (MH "Surgery, Gynecologic+") OR (MH "Surgery, Urogenital+")
2. "colorectal surgery" OR "digestive system surgical procedures" OR "gynecologic surgery OR urologic surgical procedures, male OR urologic surgical procedures" OR "biliary tract surgical procedures"
3. 1 or 2
4. (MH "Dexamethasone") OR (MH "Glucocorticoids+") OR (MH "Adrenal Cortex Hormones+")
5. "dexamethasone" OR "glucocorticoid" OR "dexamethasone isonicotinate" OR "glucocorticoids" OR "adrenal cortex hormones" OR "adrenal cortex hormone"
6. 4 or 5
7. (MH "Pain+") OR (MH "Pelvic Pain+") OR (MH "Nociceptive Pain+") OR (MH "Pain Measurement") OR (MH "Treatment Related Pain") OR (MH "Postoperative Pain") OR (MH "Abdominal Pain+") OR (MH "Wong-Baker FACES Pain Rating Scale") OR (MH "Acute Pain (Saba CCC)") OR (MH "Pain, Procedural") OR (MH "Visceral Pain") OR (MH "Nausea and Vomiting+") OR (MH "Vomiting+") OR (MH "Nausea (Saba CCC)") OR (MH "Vomiting (Saba CCC)") OR (MH "Nausea")
8. "pain" OR "nociceptive pain" OR "acute pain" OR "pain procedural" OR "pain measurement" OR "treatment related pain" OR "postoperative pain" OR "abdominal pain" OR "visceral pain" OR "postoperative nausea and vomiting" OR "vomiting" OR "nausea and vomiting" OR "nausea"
9. 7 or 8
10. 3 or 6 or 9

**Web of Science**

1. ts=('colorectal surger* or digestive system surgical procedure* or gyn?ecolog* surgery or gyn?ecolog* operation or gyn?ecolog* surgical procedure* or operative gyn?ecolog* or uro* surger* or uro* operation or uro* surgical procedure* or urogen* surgery or urogen* tract surgery or urogen* surg* procedure* or genitour* surgery) *Indexes=SCI-EXPANDED, SSCI, A&HCI, CPCI-S, CPCI-SSH, BKCI-S, BKCI-SSH, ESCI, CCR-EXPANDED, IC Timespan=All years*
2. ts=(dexameth* or glucocort* or corticoster* or adrenal cortex hormone*) *Indexes=SCI-EXPANDED, SSCI, A&HCI, CPCI-S, CPCI-SSH, BKCI-S, BKCI-SSH, ESCI, CCR-EXPANDED, IC Timespan=All years*
3. ts=(postoperative NEAR/3 nausea NEAR/3 vomiting or vomit* or postoperative nausea and vomiting or nausea or 'nausea and vomiting') *Indexes=SCI-EXPANDED, SSCI, A&HCI, CPCI-S, CPCI-SSH, BKCI-S, BKCI-SSH, ESCI, CCR-EXPANDED, IC Timespan=All years*
4. ts=(pain* or acute NEAR/3 pain* or postoperative NEAR/3 pain or pain measurement or abdominal pain* or visceral pain* or nociceptive pain* or procedural NEAR/3 pain*) *Indexes=SCI-EXPANDED, SSCI, A&HCI, CPCI-S, CPCI-SSH, BKCI-S, BKCI-SSH, ESCI, CCR-EXPANDED, IC Timespan=All years*
5. 3 or 4
6. 1 and 2 and 5

**ClinicalTrials.gov, no filters applied**

(gastrointestinal surgery OR gynaecological surgery OR urological surgery OR digestive system surgical procedures) AND (dexamethasone OR glucocorticoids OR corticosteroids OR adrenal cortex hormones)

**ISRCTN registry, no filters applied**

("colorectal surgery" OR "gynaecological surgery" OR "digestive system surgical procedures" OR "urological surgery") AND (dexamethasone OR glucocorticoids OR corticosteroids OR "adrenal cortex hormones")

**UK Clinical Trials Gateway (UKCTG)**

Searches for dexamethasone, postoperative pain, pain, abdominal pain, pelvic pain, abdominal surgery

**WHO International Clinical Trials Registry Platform (WHO ICTRP)**

“digestive system surgical procedures” OR “colorectal surgery” OR “urological surgery” OR “gynaecological surgery”

AND

Glucocorticoid OR corticosteroid OR dexamethasone OR "adrenal cortex hormone"

AND

Pain OR “postoperative pain” OR “acute pain” OR “procedural pain” OR “abdominal pain” OR “visceral pain” OR “nocioceptive pain” OR nausea OR vomiting OR “postoperative nausea and vomiting”
