# Supplementary material for: What is the impact of dexamethasone on postoperative pain in adults undergoing general anaesthesia for elective abdominal surgery: a systematic review and meta-analysis
Source: Perioper Med (Lond). 2022 Mar 24;11:13. doi: 10.1186/s13741-022-00243-6 (PMC8942613; doi:10.1186/s13741-022-00243-6)
Supplement: Supplementary file 2 — Additional file 2. List of included items on data extraction form and relevant dropdown list. Dropdown lists were used when possible to reduce the amount of free text. [file 13741_2022_243_MOESM2_ESM.docx]

**Additional file 2**

List of included items on data extraction form and relevant dropdown list. Dropdown lists were used when possible to reduce the amount of free text.

1. Study identifier - Author Year
2. Review CM, SJC
3. Date data extracted
4. Country
5. Citation
6. Type of publication – Journal, abstract, short communication
7. Aim/ Objectives of study
8. Primary outcome
9. Secondary outcome
10. Inclusion criteria
11. Type of surgery
12. Participant characteristics
13. Number of participants randomized
14. Number of dropouts and reason
15. Number of intervention patients
16. Number of control patients
17. Type of subgroup
18. Number in subgroup
19. Type of anaesthesia – General Anaesthesia/ Regional anaesthesia/ Local Anaesthesia/ Multimodal Analgesia
20. Use of central neuraxial blockade (CNB) – Yes, No
21. Type of CNB – Spinal, Epidural, CSE
22. CNB opioids – Yes, No, Unknown
23. Type opioids
24. Use of regional anaesthesia (RA) – Yes, No
25. Type of RA
26. Dexamethasone number of doses
27. Dexamethasone dose
28. Dexamethasone timing – Preinduction, Postinduction preincision, Intraoperative, Postoperative
29. Other study drug
30. Other study drug dose
31. Other study drug timing – Preinduction, Postinduction preincision, Intraoperative, Postoperative
32. Intravenous comparator
33. Timing of comparator – Preinduction, Postinduction preincision, Intraoperative, Preinduction + Intraoperative, Postoperative
34. All outcomes reported
35. Pain score at rest ≤4 hours
    1. Dexamethasone mean and SD
    2. Comparator mean and SD
36. Pain score on movement ≤4 hours
    1. Dexamethasone mean and SD
    2. Comparator mean and SD
37. Pain score at rest 4-24 hours
    1. Dexamethasone mean and SD
    2. Comparator mean and SD
38. Pain score on movement 4-24 hours
    1. Dexamethasone mean and SD
    2. Comparator mean and SD
39. Pain score at rest ≥24 hours
    1. Dexamethasone mean and SD
    2. Comparator mean and SD
40. Pain score on movement ≥24 hours
    1. Dexamethasone mean and SD
    2. Comparator mean and SD
41. Time to first analgesia in minutes
    1. Dexamethasone mean and SD
    2. Comparator mean and SD
42. Postoperative opioid requirements in oral morphine equivalents
    1. Dexamethasone mean and SD
    2. Comparator mean and SD
43. Time in PACU in minutes
    1. Dexamethasone mean and SD
    2. Comparator mean and SD
44. Side effects related to dexamethasone administration
45. Results and conclusions
46. Additional comments

The below section was repeated for studies containing multiple doses of dexamethasone for each group of dexamethasone

1. Number in intervention group
2. Pain score at rest ≤4 hours
   1. Dexamethasone mean and SD
   2. Comparator mean and SD
3. Pain score on movement ≤4 hours
   1. Dexamethasone mean and SD
   2. Comparator mean and SD
4. Pain score at rest 4-24 hours
   1. Dexamethasone mean and SD
   2. Comparator mean and SD
5. Pain score on movement 4-24 hours
   1. Dexamethasone mean and SD
   2. Comparator mean and SD
6. Pain score at rest ≥24 hours
   1. Dexamethasone mean and SD
   2. Comparator mean and SD
7. Pain score on movement ≥24 hours
   1. Dexamethasone mean and SD
   2. Comparator mean and SD
8. Time to first analgesia in minutes
   1. Dexamethasone mean and SD
   2. Comparator mean and SD
9. Postoperative opioid requirements in oral morphine equivalents
   1. Dexamethasone mean and SD
   2. Comparator mean and SD
10. Time in PACU in minutes
    1. Dexamethasone mean and SD
    2. Comparator mean and SD
