# Supplementary material for: What is the impact of dexamethasone on postoperative pain in adults undergoing general anaesthesia for elective abdominal surgery: a systematic review and meta-analysis
Source: Perioper Med (Lond). 2022 Mar 24;11:13. doi: 10.1186/s13741-022-00243-6 (PMC8942613; doi:10.1186/s13741-022-00243-6)
Supplement: Supplementary file 3 — Additional file 3: Supplementary Table 1. Table of morphine equivalents used to convert from intravenous or oral opioids to oral morphine [file 13741_2022_243_MOESM3_ESM.docx]

Supplementary Table 1 Table of morphine equivalents used to convert from intravenous or oral opioids to oral morphine

| Drug | Potency | Equivalent to 10mg oral Morphine |
| --- | --- | --- |
| Fentanyl IV | 300 | 33mcg |
| Morphine IV | 3 | 3.3mg |
| Codeine phosphate | 0.1 | 100mg |
| Dihydrocodeine | 0.1 | 100mg |
| Hydromorphone | 7.5 | 1.3mg |
| Oxycodone PO | 2 | 5mg |
| Oxycodone IV | 3 | 3.3mg |
| Tapentadol | 0.4 | 25mg |
| Tramadol PO | 0.15 | 67mg |
| Tramadol IV | 0.3 | 33mg |
| Meperidine IV  (Pethidine) | 0.4 | 25mg |
| Nalbuphine | 3 | 3.3mg |

Faculty of Pain Medicine. Dose equivalents and changing opioids [Internet]. London. [cited 2020 March 23]. Available from: https://fpm.ac.uk/opioids-aware-structured-approach-opioid-prescribing/dose-equivalents-and-changing-opioids
