# Supplementary material for: What is the impact of dexamethasone on postoperative pain in adults undergoing general anaesthesia for elective abdominal surgery: a systematic review and meta-analysis
Source: Perioper Med (Lond). 2022 Mar 24;11:13. doi: 10.1186/s13741-022-00243-6 (PMC8942613; doi:10.1186/s13741-022-00243-6)
Supplement: Supplementary file 4 — Additional file 4: Supplementary Table 2. Tabular Risk of Bias Assessment [file 13741_2022_243_MOESM4_ESM.docx]

Supplementary Table 2 Tabular Risk of Bias Assessment

| **Study** | Alghanem 2010 | |
| --- | --- | --- |
| **Domain being assessed** | VAS Pain | |
| **Risk of Bias Domain** | **Judgement** | **Support for judgement** |
| Randomisation process | Low | Syringes were prepared by the pharmacist according to a table of randomised numbers.  No baseline differences between groups. |
| Deviation from the intended interventions | Low | Blinding was used.  Appropriate post randomisation exclusions. |
| Missing outcome data | Low | Outcome data of 100% of post randomisation excluded patients are available. |
| Measurement of the outcome | Low | Outcome assessments done at the same time points and frequency.  Outcome assessors blinded. |
| Selection of the reported result | Some concerns | VAS recorded and reported at 4 and 24 hours with no statistical significance. |
| Overall risk of bias | Some concerns |  |

| **Study** | Areeruk 2016 | |
| --- | --- | --- |
| **Domain being assessed** | NRS Pain | |
| **Risk of Bias Domain** | **Judgement** | **Support for judgement** |
| Randomisation process | Low | Computer generated random number list. Study solution prepared by nurse not involved and stored in a sealed envelope and given to an anaesthetist not involved.  No baseline differences between groups. |
| Deviation from the intended interventions | High | Blinding through use of placebo.  3/52 patients excluded post randomisation for reasons not prespecified. |
| Missing outcome data | Low | Outcome data available 49/52 randomised |
| Measurement of the outcome | Low | Outcome assessments done at the same time points and frequency.  Outcome assessors blinded. |
| Selection of the reported result | Low | Outcomes presented as prespecified in trial protocol. |
| Overall risk of bias | High |  |

| **Study** | Badawy 2015 | |
| --- | --- | --- |
| **Domain being assessed** | VAS Pain | |
| **Risk of Bias Domain** | **Judgement** | **Support for judgement** |
| Randomisation process | Some concerns | Randomly allocated using closed envelope technique.  Minimal information on allocation concealment.  No baseline differences between groups. |
| Deviation from the intended interventions | Low | Blinding through use of placebo.  No post randomisation exclusions. 3/60 lost to follow-up |
| Missing outcome data | Low | Outcome data available 57/60 randomised. |
| Measurement of the outcome | Low | Outcome assessments done at the same time points and frequency.  Outcome assessors blinded. |
| Selection of the reported result | Low | Outcomes reported as prespecified in methods. |
| Overall risk of bias | Some concerns |  |

| **Study** | Bataille 2016 | |
| --- | --- | --- |
| **Domain being assessed** | VRS Pain | |
| **Risk of Bias Domain** | **Judgement** | **Support for judgement** |
| Randomisation process | Low | Internet-based randomisation system in blocks of 10.  Centrally prepared identical drugs.  No baseline differences between groups. |
| Deviation from the intended interventions | Some concerns | Blinding through use of placebo.  Per-protocol analysis |
| Missing outcome data | Low | Outcome data available 117/122 randomised. |
| Measurement of the outcome | Low | Outcome assessment the same between groups.  Outcome assessors blinded. |
| Selection of the reported result | Some concerns | Minimal information on outcome assessment and measurement in trial registry. |
| Overall risk of bias | Some concerns |  |

| **Study** | Batistaki 2019 | |
| --- | --- | --- |
| **Domain being assessed** | NRS Pain | |
| **Risk of Bias Domain** | **Judgement** | **Support for judgement** |
| Randomisation process | High | Computer-generated randomisation.  Lack of allocation concealment.  Baseline differences between groups likely due to chance. |
| Deviation from the intended interventions | Low | Blinding through use of placebo.  Modified ITT analysis |
| Missing outcome data | Some concerns | Outcome data available 44/50 randomised. |
| Measurement of the outcome | Low | Outcome assessment the same between groups.  Outcome assessors blinded. |
| Selection of the reported result | Low | Outcomes presented as prespecified in trial protocol. |
| Overall risk of bias | High |  |

| **Study** | Benevides 2013 | |
| --- | --- | --- |
| **Domain being assessed** | Opioid consumption | |
| **Risk of Bias Domain** | **Judgement** | **Support for judgement** |
| Randomisation process | Low | Computer-generated randomisation. Sealed envelopes containing the groups opened in the operating room.  No baseline differences between groups. |
| Deviation from the intended interventions | Some concerns | Blinding through use of placebo.  Per-protocol analysis |
| Missing outcome data | Low | Outcome data available 90/96 randomised. |
| Measurement of the outcome | Low | Outcome assessment the same between groups.  Outcome assessors blinded. |
| Selection of the reported result | Low | Outcomes presented as prespecified in trial protocol. |
| Overall risk of bias | Some concerns |  |

| **Study** | Bianchin 2007 | |
| --- | --- | --- |
| **Domain being assessed** | VAS Pain | |
| **Risk of Bias Domain** | **Judgement** | **Support for judgement** |
| Randomisation process | Some concerns | Computer-generated random number table.  No information on allocation concealment.  No baseline differences between groups. |
| Deviation from the intended interventions | High | Blinding through use of placebo.  7/80 patients excluded post randomisation for reasons not prespecified |
| Missing outcome data | Low | Outcome data available 73/80 randomised.  Reasons for missing data documented and unrelated to outcome. |
| Measurement of the outcome | Low | Outcome assessments done at the same time points and frequency.  Outcome assessors blinded. |
| Selection of the reported result | Some concerns | Minimal information on reporting and analysis plan. |
| Overall risk of bias | High |  |

| **Study** | Bilgin 2010 | |
| --- | --- | --- |
| **Domain being assessed** | VAS Pain | |
| **Risk of Bias Domain** | **Judgement** | **Support for judgement** |
| Randomisation process | Low | Computer-generated random number table.  Study drugs prepared into identical containers.  No baseline differences between groups. |
| Deviation from the intended interventions | Low | Blinding through use of placebo. |
| Missing outcome data | Low | Outcome data available for all 120 randomised. |
| Measurement of the outcome | Low | Outcome assessments done at the same time points and frequency.  Outcome assessors blinded. |
| Selection of the reported result | High | Not all prespecified pain scores presented. Lack of statistical significance |
| Overall risk of bias | High |  |

| **Study** | Bisgaard 2003 | |
| --- | --- | --- |
| **Domain being assessed** | VAS Pain | |
| **Risk of Bias Domain** | **Judgement** | **Support for judgement** |
| Randomisation process | Low | Computer-generated block randomisation in sealed envelope.  Randomisation concealed until study complete.  No baseline differences between groups. |
| Deviation from the intended interventions | Low | Blinding through use of placebo.  Appropriate post randomisation exclusions.  m-ITT single participant withdrew. |
| Missing outcome data | Some concerns | Outcome data available for 80/88 randomised. |
| Measurement of the outcome | Low | Outcome assessments done at the same time points and frequency.  Outcome assessors blinded. |
| Selection of the reported result | High | Appears to be multiple analysis of data.  Non-significant results not reported. |
| Overall risk of bias | High |  |

| **Study** | Coloma 2002 | |
| --- | --- | --- |
| **Domain being assessed** | Opioid consumption | |
| **Risk of Bias Domain** | **Judgement** | **Support for judgement** |
| Randomisation process | Some concerns | Randomly assigned; minimal information on randomisation or allocation concealment.  No baseline differences between groups. |
| Deviation from the intended interventions | Low | Blinding through use of placebo. |
| Missing outcome data | Low | Outcome assessments available for all 140 randomised. |
| Measurement of the outcome | Low | Outcome assessment done at same time point.  Outcome assessors blinded. |
| Selection of the reported result | Low | Outcome presented as specified in methods. |
| Overall risk of bias | Some concerns |  |

| **Study** | Corcoran 2017 | |
| --- | --- | --- |
| **Domain being assessed** | VAS Pain | |
| **Risk of Bias Domain** | **Judgement** | **Support for judgement** |
| Randomisation process | Low | Computer-generated random number sequence allocated using sealed opaque envelopes.  Study drug prepared by external observer.  No baseline differences in groups. |
| Deviation from the intended interventions | Some concerns | Blinding through use of placebo.  Per-protocol analysis. |
| Missing outcome data | Low | Outcome data available for 31/32 randomised. |
| Measurement of the outcome | Low | Outcome assessment done at same time point and frequency.  Outcome assessors blinded. |
| Selection of the reported result | High | Secondary outcomes and analysis plan not prespecified in trial registry. Outcome presented not as documented in methods. |
| Overall risk of bias | High |  |

| **Study** | De Oliveira 2011 | |
| --- | --- | --- |
| **Domain being assessed** | NRS Pain | |
| **Risk of Bias Domain** | **Judgement** | **Support for judgement** |
| Randomisation process | Low | Computer-generated randomly assigned in sequentially numbered opaque envelopes.  No baseline differences between groups. |
| Deviation from the intended interventions | Low | Blinding through use of placebo.  Modified intention to treat analysis. |
| Missing outcome data | Some concerns | Outcome data available for 106/120 randomised.  Reasons for missing outcomes documented, could depend on true value. |
| Measurement of the outcome | Low | Outcome assessment done at same time point and frequency.  Outcome assessors blinded. |
| Selection of the reported result | High | Outcome measured at regular intervals but presented at two. |
| Overall risk of bias | High |  |

| **Study** | Elhakim 2002 | |
| --- | --- | --- |
| **Domain being assessed** | VAS Pain | |
| **Risk of Bias Domain** | **Judgement** | **Support for judgement** |
| Randomisation process | Some concerns | Computer-generated random number table.  No information on allocation concealment.  No baseline differences between groups. |
| Deviation from the intended interventions | Low | Blinding through use of placebo.  No post randomisation exclusions. |
| Missing outcome data | Low | Outcome data available for all 180 randomised. |
| Measurement of the outcome | Low | Outcome assessment done at same time point and frequency.  Outcome assessors blinded. |
| Selection of the reported result | High | Multiple outcome measures not presented as specified in methods. Lack of statistical significance. |
| Overall risk of bias | High |  |

| **Study** | Feo 2006 | |
| --- | --- | --- |
| **Domain being assessed** | VAS Pain | |
| **Risk of Bias Domain** | **Judgement** | **Support for judgement** |
| Randomisation process | Some concerns | Randomly assigned; minimal information on randomisation or allocation concealment.  No baseline differences between groups. |
| Deviation from the intended interventions | Low | Blinding through use of placebo. |
| Missing outcome data | Low | Outcome data available for all 101 randomised. |
| Measurement of the outcome | Low | Outcome assessment done at same time point and frequency.  Outcome assessors blinded. |
| Selection of the reported result | Low | Outcome presented as specified in methods. |
| Overall risk of bias | Some concerns |  |

| **Study** | Fukami 2009 | |
| --- | --- | --- |
| **Domain being assessed** | VAS Pain | |
| **Risk of Bias Domain** | **Judgement** | **Support for judgement** |
| Randomisation process | Some concerns | Randomised using blind envelopes.  No information regarding allocation concealment.  No baseline differences between groups. |
| Deviation from the intended interventions | Some concerns | Blinding through use of placebo.  1/88 post randomisation exclusion for reasons not prespecified |
| Missing outcome data | Low | Outcome data available for 80/88 randomised.  Reasons clearly documented |
| Measurement of the outcome | Low | Outcome assessment done at same time point and frequency.  Outcome assessors blinded. |
| Selection of the reported result | Low | Outcome presented and analysed as specified in methods. |
| Overall risk of bias | Some concerns |  |

| **Study** | Gautam 2008 | |
| --- | --- | --- |
| **Domain being assessed** | VAS Pain | |
| **Risk of Bias Domain** | **Judgement** | **Support for judgement** |
| Randomisation process | Low | Computer-generated random number table.  Identical syringes prepared by personnel not involved in the study.  No baseline differences between groups. |
| Deviation from the intended interventions | Low | Blinding through use of placebo.  8/150 appropriate post randomisation exclusions |
| Missing outcome data | Low | Outcome data available for 142/150 randomised for appropriate post randomisation exclusions. |
| Measurement of the outcome | Low | Outcome assessment done at same time point and frequency.  Outcome assessors blinded. |
| Selection of the reported result | Low | Outcome presented and analysed as specified in methods. All non-significant results reported |
| Overall risk of bias | Low |  |

| **Study** | Hammas 2002 | |
| --- | --- | --- |
| **Domain being assessed** | VAS Pain | |
| **Risk of Bias Domain** | **Judgement** | **Support for judgement** |
| Randomisation process | Low | Stratified randomisation using concealed envelopes.  No baseline differences between groups. |
| Deviation from the intended interventions | Some concerns | Participants and caregiver unblinded.  8/180 protocol violations could be consistent with what would occur outside trial context.  Per-protocol analysis |
| Missing outcome data | Low | Outcome data available for 172/180 randomised. |
| Measurement of the outcome | Some concerns | Outcome assessors were unblinded and subjective outcome. |
| Selection of the reported result | High | Outcome measured but not reported. |
| Overall risk of bias | High |  |

| **Study** | Ionescu 2014 | |
| --- | --- | --- |
| **Domain being assessed** | Opioid consumption | |
| **Risk of Bias Domain** | **Judgement** | **Support for judgement** |
| Randomisation process | Some concerns | Computer-generated random sequence.  Minimal information regarding allocation concealment.  No baseline differences between groups. |
| Deviation from the intended interventions | High | Blinding through use of placebo.  Post-randomisation exclusions in 3/46 for unexplained technical reasons. |
| Missing outcome data | Low | Outcome data available for 42/46 randomised.  Missingness of outcome data likely unrelated to outcome. |
| Measurement of the outcome | Low | Outcome assessment done at same time point.  Outcome assessors blinded. |
| Selection of the reported result | Low | Outcome presented as specified in methods. |
| Overall risk of bias | High |  |

| **Study** | Jo 2012 | |
| --- | --- | --- |
| **Domain being assessed** | VAS Pain | |
| **Risk of Bias Domain** | **Judgement** | **Support for judgement** |
| Randomisation process | Low | Computer-generated randomisation placed in sealed envelope and opened by independent physician prior to induction of anaesthesia.  No baseline differences between groups. |
| Deviation from the intended interventions | Low | Blinding through use of placebo. |
| Missing outcome data | Low | Outcome data available for 120/120 randomised. |
| Measurement of the outcome | Low | Outcome assessment done at same time point and frequency.  Outcome assessors blinded. |
| Selection of the reported result | Some concerns | Minimal prespecified information on outcome measurement.  Non-significant outcomes reported. |
| Overall risk of bias | Some concerns |  |

| **Study** | Jokela 2009 | |
| --- | --- | --- |
| **Domain being assessed** | VAS Pain | |
| **Risk of Bias Domain** | **Judgement** | **Support for judgement** |
| Randomisation process | Low | Computer-generated random number sequence performed centrally. Each patient allocated with a consecutive randomisation number.  No baseline differences between groups. |
| Deviation from the intended interventions | High | Blinding through use of placebo.  Per-protocol analysis with 9/129 post-randomisation exclusions |
| Missing outcome data | Low | Outcome data available for 120/129 randomised.  Documented reasons for all missing data. |
| Measurement of the outcome | Low | Outcome assessment done at same time point and frequency.  Outcome assessors blinded. |
| Selection of the reported result | Low | Outcome presented as specified in methods. |
| Overall risk of bias | High |  |

| **Study** | Kasagi 2013 | |
| --- | --- | --- |
| **Domain being assessed** | VAS Pain | |
| **Risk of Bias Domain** | **Judgement** | **Support for judgement** |
| Randomisation process | Some concerns | Randomised by opaque envelope method.  Minimal information regarding allocation concealment.  No baseline differences between groups. |
| Deviation from the intended interventions | Low | Patients and outcome assessors blinded.  Anaesthetist responsible for intraoperative care not blinded. |
| Missing outcome data | Low | Outcome data available for all 120 randomised. |
| Measurement of the outcome | Low | Outcome assessment done at same time point and frequency.  Outcome assessors blinded. |
| Selection of the reported result | Low | Outcome presented as specified in methods. |
| Overall risk of bias | Some concerns |  |

| **Study** | Kassim 2018 | |
| --- | --- | --- |
| **Domain being assessed** | VAS Pain | |
| **Risk of Bias Domain** | **Judgement** | **Support for judgement** |
| Randomisation process | Low | Computer-generated random table using sequentially numbered, opaque, sealed envelopes.  Study drugs prepared by hospital pharmacy and indistinguishable.  No baseline differences between groups. |
| Deviation from the intended interventions | Low | Blinding through use of placebo. |
| Missing outcome data | Low | Outcome data available for 75 randomised. |
| Measurement of the outcome | Low | Outcome assessment done at same time point and frequency.  Outcome assessors blinded. |
| Selection of the reported result | Low | Outcome presented as prespecified in trial registry. |
| Overall risk of bias | Low |  |

| **Study** | Ko-iam 2015 | |
| --- | --- | --- |
| **Domain being assessed** | NRS Pain | |
| **Risk of Bias Domain** | **Judgement** | **Support for judgement** |
| Randomisation process | Low | Block of four randomisation by hospital pharmacist.  Concealment by opaque envelopes opened before the end of surgery and blinded syringes supplied by pharmacist.  No baseline differences between groups. |
| Deviation from the intended interventions | Low | Blinding through use of placebo. |
| Missing outcome data | Low | Outcome data available for all 100 randomised. |
| Measurement of the outcome | Low | Outcome assessment done at same time point and frequency.  Outcome assessors blinded. |
| Selection of the reported result | High | One pain score presented but multiple measurements. Lack of statistical significance. |
| Overall risk of bias | High |  |

| **Study** | Kurz 2015 | |
| --- | --- | --- |
| **Domain being assessed** | VAS Pain | |
| **Risk of Bias Domain** | **Judgement** | **Support for judgement** |
| Randomisation process | Low | Computer-generated random sequence in an opaque sealed envelope.  Study drugs independently prepared in identical format.  No baseline differences between groups. |
| Deviation from the intended interventions | Some concerns | Blinding through use of placebo. Anaesthetist not blinded. Recorded sealed after PACU handoff.  Per-protocol analysis |
| Missing outcome data | Low | Outcome data available for 555/586 randomised. |
| Measurement of the outcome | Low | Outcome assessment done at same time point and frequency.  Outcome assessors blinded. |
| Selection of the reported result | High | Multiple outcome measures taken but not reported. |
| Overall risk of bias | High |  |

| **Study** | Lee 2017 | |
| --- | --- | --- |
| **Domain being assessed** | VAS Pain | |
| **Risk of Bias Domain** | **Judgement** | **Support for judgement** |
| Randomisation process | Some concerns | Randomised using sealed envelopes.  No information on allocation concealment  Baseline differences between groups compatible with chance. |
| Deviation from the intended interventions | Some concerns | Blinding through use of placebo.  Per-protocol analysis with 12/392 post-randomisation exclusions |
| Missing outcome data | Low | Outcome data available for 380/392 randomised. |
| Measurement of the outcome | Low | Outcome assessment done at same time point and frequency.  Outcome assessors blinded. |
| Selection of the reported result | Low | Outcome presented as specified in methods. |
| Overall risk of bias | Some concerns |  |

| **Study** | Lim 2011 | |
| --- | --- | --- |
| **Domain being assessed** | VAS Pain | |
| **Risk of Bias Domain** | **Judgement** | **Support for judgement** |
| Randomisation process | Some concerns | Minimal information on randomisation or allocation concealment.  No baseline differences between groups. |
| Deviation from the intended interventions | Low | Blinding through use of placebo. |
| Missing outcome data | Low | Outcome data available for 120 randomised. |
| Measurement of the outcome | Low | Outcome assessment done at same time point and frequency.  Outcome assessors blinded. |
| Selection of the reported result | Low | Outcome presented as specified in methods. |
| Overall risk of bias | Some concerns |  |

| **Study** | Liu 1998 | |
| --- | --- | --- |
| **Domain being assessed** | VRS Pain | |
| **Risk of Bias Domain** | **Judgement** | **Support for judgement** |
| Randomisation process | Some concerns | No information regarding randomisation or allocation concealment.  No baseline differences between groups. |
| Deviation from the intended interventions | Some concerns | Blinding through use of placebo.  Unclear if patients analysed in groups that they were randomised. |
| Missing outcome data | High | Outcome data not available for this outcome.  No information on missing outcome data. |
| Measurement of the outcome | Low | Outcome assessment done at same time point and frequency.  Outcome assessors blinded. |
| Selection of the reported result | High | Outcome data not presented as specified in methods. Lack of statistical significance |
| Overall risk of bias | High |  |

| **Study** | Liu 1999 | |
| --- | --- | --- |
| **Domain being assessed** | VAS Pain | |
| **Risk of Bias Domain** | **Judgement** | **Support for judgement** |
| Randomisation process | Some concerns | No information on randomisation or allocation concealment.  No baseline differences between groups. |
| Deviation from the intended interventions | Low | Blinding through use of placebo. |
| Missing outcome data | Some concerns | No information on numbers of outcome data. |
| Measurement of the outcome | Low | Outcome assessment done at same time point and frequency.  Outcome assessors blinded. |
| Selection of the reported result | High | Multiple outcome measures recorded but not presented as specified in methods. No statistical significance. |
| Overall risk of bias | High |  |

| **Study** | López-Olaondo 1996 | |
| --- | --- | --- |
| **Domain being assessed** | VAS Pain | |
| **Risk of Bias Domain** | **Judgement** | **Support for judgement** |
| Randomisation process | Some concerns | Minimal information regarding randomisation and allocation concealment.  No baseline differences between groups. |
| Deviation from the intended interventions | Low | Blinding through use of placebo. |
| Missing outcome data | Low | Outcome data available for 100 randomised. |
| Measurement of the outcome | Low | Outcome assessment done at same time point and frequency.  Outcome assessors blinded. |
| Selection of the reported result | Low | Outcome presented as prespecified |
| Overall risk of bias | Some concerns |  |

| **Study** | Maddali 2003 | |
| --- | --- | --- |
| **Domain being assessed** | VAS Pain | |
| **Risk of Bias Domain** | **Judgement** | **Support for judgement** |
| Randomisation process | Some concerns | Random number table.  Minimal information on allocation concealment.  No baseline differences between groups. |
| Deviation from the intended interventions | Low | Blinding through use of placebo. |
| Missing outcome data | Low | Outcome data available for all 120 randomised. |
| Measurement of the outcome | Low | Outcome assessment done at same time point and frequency.  Outcome assessors blinded. |
| Selection of the reported result | High | Minimal information on planned analysis of outcome.  Selective outcome reporting. |
| Overall risk of bias | High |  |

| **Study** | Mathiesen 2009 | |
| --- | --- | --- |
| **Domain being assessed** | VAS Pain | |
| **Risk of Bias Domain** | **Judgement** | **Support for judgement** |
| Randomisation process | Low | Computer-generated block randomisation.  Centrally prepared sequentially labelled study drugs.  No baseline differences between groups. |
| Deviation from the intended interventions | High | Blinding through use of placebo.  12/128 post randomisation exclusion. Per protocol analysis |
| Missing outcome data | Some concerns | Outcome data available for 116/128 randomised.  Missingness of outcome data could be related to its true value. |
| Measurement of the outcome | Low | Outcome assessment done at same time point and frequency.  Outcome assessors blinded. |
| Selection of the reported result | Low | Outcome presented as prespecified in trial registry. |
| Overall risk of bias | High |  |

| **Study** | McKenzie 1997 | |
| --- | --- | --- |
| **Domain being assessed** | VAS Pain | |
| **Risk of Bias Domain** | **Judgement** | **Support for judgement** |
| Randomisation process | Low | Stratified randomisation via random number table.  Independently prepared identical study drugs.  No baseline differences between groups. |
| Deviation from the intended interventions | Low | Blinding through use of placebo. |
| Missing outcome data | Low | Outcome data available for all 80 randomised. |
| Measurement of the outcome | Low | Outcome assessment done at same time point and frequency.  Outcome assessors blinded. |
| Selection of the reported result | Low | Outcome presented as specified in methods. |
| Overall risk of bias | Low |  |

| **Study** | Murphy 2011 | |
| --- | --- | --- |
| **Domain being assessed** | VAS Pain | |
| **Risk of Bias Domain** | **Judgement** | **Support for judgement** |
| Randomisation process | Low | Computer-generated random sequence controlled centrally.  Study drugs centrally prepared in identical syringes.  Baseline difference between groups likely due to chance. |
| Deviation from the intended interventions | Low | Blinding through use of placebo.  No inappropriate post randomisation exclusions. |
| Missing outcome data | Low | Outcome data available for 115/120 randomised. |
| Measurement of the outcome | Low | Outcome assessment done at same time point and frequency.  Outcome assessors blinded. |
| Selection of the reported result | Low | Outcome presented as specified in methods.  Lack of statistical significance. |
| Overall risk of bias | Low |  |

| **Study** | Murphy 2014 | |
| --- | --- | --- |
| **Domain being assessed** | VAS Pain | |
| **Risk of Bias Domain** | **Judgement** | **Support for judgement** |
| Randomisation process | Low | Computer-generated block randomisation.  Centrally stored randomisation codes.  Study drugs centrally prepared in identical syringes.  No baseline differences between groups. |
| Deviation from the intended interventions | Some concerns | Blinding through use of placebo.  Post randomisation exclusion for protocol violation. |
| Missing outcome data | Low | Outcome data available for 195/200 randomised. |
| Measurement of the outcome | Low | Outcome assessment done at same time point and frequency.  Outcome assessors blinded. |
| Selection of the reported result | Low | Outcome presented as specified in methods. |
| Overall risk of bias | Some concerns |  |

| **Study** | Nesek-Adam 2007 | |
| --- | --- | --- |
| **Domain being assessed** | VAS Pain | |
| **Risk of Bias Domain** | **Judgement** | **Support for judgement** |
| Randomisation process | Some concerns | Minimal information on randomisation or allocation concealment.  No baseline differences between groups. |
| Deviation from the intended interventions | Low | Blinding through use of placebo. |
| Missing outcome data | Low | Outcome data available for all 120 randomised. |
| Measurement of the outcome | Low | Outcome assessment done at same time point and frequency.  Outcome assessors blinded. |
| Selection of the reported result | Some concerns | Outcome not completely presented as specified in methods.  Lack of statistical significance |
| Overall risk of bias | Some concerns |  |

| **Study** | Olajumoke 2013 | |
| --- | --- | --- |
| **Domain being assessed** | PACU LOS | |
| **Risk of Bias Domain** | **Judgement** | **Support for judgement** |
| Randomisation process | Low | Random number tables.  Labelled syringes prepared by pharmacist and anaesthetic resident with blinding of researcher.  No baseline demographics presented. |
| Deviation from the intended interventions | Some concerns | Blinding through use of placebo.  Post randomisation exclusions for reasons not prespecified |
| Missing outcome data | Low | Outcome data available for 96/100 randomised. |
| Measurement of the outcome | Low | Outcome assessment done at same time point.  Outcome assessors blinded. |
| Selection of the reported result | Some concerns | Minimal information on planned reporting and analysis of outcomes. |
| Overall risk of bias | Some concerns |  |

| **Study** | Pan 2008 | |
| --- | --- | --- |
| **Domain being assessed** | VAS Pain | |
| **Risk of Bias Domain** | **Judgement** | **Support for judgement** |
| Randomisation process | Some concerns | Computer-generated random sequence.  Minimal information on allocation concealment  No baseline differences between groups. |
| Deviation from the intended interventions | Some concerns | Blinding through use of placebo.  Post randomisation exclusions for reasons not prespecified |
| Missing outcome data | Low | Outcome data available for 60/64 randomised. |
| Measurement of the outcome | Low | Outcome assessment done at same time point and frequency.  Outcome assessors blinded. |
| Selection of the reported result | Low | Outcome presented as specified in methods.  Lack of statistical significance. |
| Overall risk of bias | Some concerns |  |

| **Study** | Pauls 2015 | |
| --- | --- | --- |
| **Domain being assessed** | VAS Pain | |
| **Risk of Bias Domain** | **Judgement** | **Support for judgement** |
| Randomisation process | Low | Computer-generated random sequence.  Centralised allocation of study drugs.  Imbalance compatible with chance. |
| Deviation from the intended interventions | High | Blinding through use of placebo.  Per-protocol analysis. |
| Missing outcome data | High | Outcome data available for 63/74 randomised resulting in significant group imbalances.  Reasons for missing data documented and could be related to the outcome. |
| Measurement of the outcome | Low | Outcome assessment done at same time point.  Outcome assessors blinded. |
| Selection of the reported result | Low | Outcome presented as specified in methods. |
| Overall risk of bias | High |  |

| **Study** | Regasa 2020 | |
| --- | --- | --- |
| **Domain being assessed** | NRS Pain | |
| **Risk of Bias Domain** | **Judgement** | **Support for judgement** |
| Randomisation process | High | Ballot box randomisation.  No allocation concealment.  No baseline differences between groups. |
| Deviation from the intended interventions | Low | Blinding through use of control. |
| Missing outcome data | High | Outcome data not available for this outcome.  No information available. |
| Measurement of the outcome | Low | Outcome assessment done at same time point.  Outcome assessors blinded. |
| Selection of the reported result | Some concerns | Outcome not presented as specified in methods. |
| Overall risk of bias | High |  |

| **Study** | Rothenberg 1998 | |
| --- | --- | --- |
| **Domain being assessed** | VAS Pain | |
| **Risk of Bias Domain** | **Judgement** | **Support for judgement** |
| Randomisation process | Low | Minimal information on randomisation.  Study drugs prepared centrally into numbered syringes of identical volumes.  No baseline differences between groups. |
| Deviation from the intended interventions | Some concerns | Blinding through use of control.  Post-randomisation exclusions for reasons not prespecified. |
| Missing outcome data | Low | Outcome data available for 95/100 randomised. |
| Measurement of the outcome | Low | Outcome assessment done at same time point and frequency.  Outcome assessors blinded. |
| Selection of the reported result | Some concerns | Minimal information on analysis plan. |
| Overall risk of bias | Some concerns |  |

| **Study** | Ryu 2013 | |
| --- | --- | --- |
| **Domain being assessed** | NRS Pain | |
| **Risk of Bias Domain** | **Judgement** | **Support for judgement** |
| Randomisation process | High | Computer-generated randomisation in block of four.  Anaesthetist performed randomisation and allocation.  No baseline differences between groups. |
| Deviation from the intended interventions | Some concerns | Blinding through use of placebo.  Per-protocol analysis |
| Missing outcome data | Low | Outcome data available for 72/76 randomised. |
| Measurement of the outcome | Low | Outcome assessment done at same time point and frequency.  Outcome assessors blinded. |
| Selection of the reported result | High | Single outcome with statistical significance presented for large time interval. Unclear how this was derived. |
| Overall risk of bias | High |  |

| **Study** | Sanchez-Ledesma 2002 | |
| --- | --- | --- |
| **Domain being assessed** | VAS Pain | |
| **Risk of Bias Domain** | **Judgement** | **Support for judgement** |
| Randomisation process | Low | Randomisation by single aleatory assignment.  Study drug centrally prepared in syringes of identical volume.  No baseline differences between groups. |
| Deviation from the intended interventions | Low | Blinding through use of control. |
| Missing outcome data | Low | Outcome data available for all 90 randomised. |
| Measurement of the outcome | Low | Outcome assessment done at same time point and frequency.  Outcome assessors blinded. |
| Selection of the reported result | Low | Outcome data presented as specified in methods. Acquired from author. |
| Overall risk of bias | Low |  |

| **Study** | Sánchez-Rodríguez 2010 | |
| --- | --- | --- |
| **Domain being assessed** | VAS Pain | |
| **Risk of Bias Domain** | **Judgement** | **Support for judgement** |
| Randomisation process | Some concerns | Randomisation using blind envelopes.  Minimal information on allocation concealment.  No baseline differences between groups. |
| Deviation from the intended interventions | Low | Blinding through use of placebo. |
| Missing outcome data | Low | Outcome data available for all 210 randomised. |
| Measurement of the outcome | Low | Outcome assessment done at same time point and frequency.  Outcome assessors blinded. |
| Selection of the reported result | Low | Outcome presented as specified in methods. |
| Overall risk of bias | Some concerns |  |

| **Study** | Shrestha 2014 | |
| --- | --- | --- |
| **Domain being assessed** | VAS Pain | |
| **Risk of Bias Domain** | **Judgement** | **Support for judgement** |
| Randomisation process | Some concerns | Computer-generated random number table.  No information on allocation concealment.  No baseline differences between groups. |
| Deviation from the intended interventions | Low | Blinding through use of placebo.  Unequal volumes of study drugs but no apparent deviations from intended interventions. |
| Missing outcome data | Low | Outcome data available for all 120 patients randomised. |
| Measurement of the outcome | Low | Outcome assessment done at same time point.  Outcome assessors blinded. |
| Selection of the reported result | Low | Outcome presented as specified in methods. |
| Overall risk of bias | Some concerns |  |

| **Study** | Sistla 2009 | |
| --- | --- | --- |
| **Domain being assessed** | VAS Pain | |
| **Risk of Bias Domain** | **Judgement** | **Support for judgement** |
| Randomisation process | Some concerns | Randomisation via sealed envelope.  Minimal information on allocation concealment.  No baseline differences between groups. |
| Deviation from the intended interventions | Some concerns | Blinding through use of placebo.  Post randomisation exclusions for reasons not prespecified. |
| Missing outcome data | High | Outcome data available for 70/79 randomised.  Missingness of data likely depends on true value. |
| Measurement of the outcome | Low | Outcome assessment done at same time point and frequency.  Outcome assessors blinded. |
| Selection of the reported result | Low | Outcome presented as specified in methods. |
| Overall risk of bias | High |  |

| **Study** | Thangaswamy 2010 | |
| --- | --- | --- |
| **Domain being assessed** | VAS Pain | |
| **Risk of Bias Domain** | **Judgement** | **Support for judgement** |
| Randomisation process | Low | Computer-generated random number list.  Numbered study solution kept in sealed envelope.  No baseline differences between groups. |
| Deviation from the intended interventions | Low | Blinding through use of placebo. |
| Missing outcome data | Low | Outcome data available for all 55 randomised. |
| Measurement of the outcome | Low | Outcome assessment done at same time point and frequency.  Outcome assessors blinded. |
| Selection of the reported result | Low | Outcome presented as specified in methods. |
| Overall risk of bias | Low |  |

| **Study** | Tolver 2012 | |
| --- | --- | --- |
| **Domain being assessed** | VAS Pain | |
| **Risk of Bias Domain** | **Judgement** | **Support for judgement** |
| Randomisation process | Low | Computer-generated block randomisation.  Study drugs contained in numbered opaque sealed envelopes until allocated.  No baseline differences between groups. |
| Deviation from the intended interventions | Low | Blinding through use of placebo.  Modified intention-to-treat analysis. |
| Missing outcome data | Low | Outcome data available for 73/80 randomised.  Reasons documented for missing data and likely unrelated to outcome. |
| Measurement of the outcome | Low | Outcome assessment done at same time point and frequency.  Outcome assessors blinded. |
| Selection of the reported result | Low | Multiple analysis performed and presented as specified in statistical analysis plan. |
| Overall risk of bias | Low |  |

| **Study** | Viriyaroj 2008 | |
| --- | --- | --- |
| **Domain being assessed** | VAS Pain | |
| **Risk of Bias Domain** | **Judgement** | **Support for judgement** |
| Randomisation process | Some concerns | Randomisation using sealed envelope technique.  Minimal information on allocation concealment.  No baseline differences between groups. |
| Deviation from the intended interventions | Low | Blinding through use of placebo. |
| Missing outcome data | Low | Outcome data available for all 80 randomised. |
| Measurement of the outcome | Low | Outcome assessment done at same time point and frequency.  Outcome assessors blinded. |
| Selection of the reported result | Some concerns | Minimal information on prespecified statistical analysis plan. |
| Overall risk of bias | Some concerns |  |

| **Study** | Wang 1999 | |
| --- | --- | --- |
| **Domain being assessed** | VAS Pain | |
| **Risk of Bias Domain** | **Judgement** | **Support for judgement** |
| Randomisation process | Some concerns | Computer-generated random sequence.  Minimal information on allocation concealment.  No baseline differences between groups. |
| Deviation from the intended interventions | High | Blinding through use of placebo.  Post-randomisation exclusions for reasons not prespecified. |
| Missing outcome data | Some concerns | Outcome data available for 78/90 randomised.  Reasons for missing outcome data could depend on the true value but evenly distributed between groups. |
| Measurement of the outcome | Low | Outcome assessment done at same time point and frequency.  Outcome assessors blinded. |
| Selection of the reported result | Low | Outcome presented as specified in methods. |
| Overall risk of bias | High |  |

| **Study** | Wang 2000 | |
| --- | --- | --- |
| **Domain being assessed** | VAS Pain | |
| **Risk of Bias Domain** | **Judgement** | **Support for judgement** |
| Randomisation process | High | Computer-generated random sequence.  Probably inadequate allocation concealment  No baseline differences between groups. |
| Deviation from the intended interventions | Low | Blinding through use of placebo. |
| Missing outcome data | Low | Outcome data available for 120 randomised. |
| Measurement of the outcome | Low | Outcome assessment done at same time point and frequency.  Outcome assessors blinded. |
| Selection of the reported result | High | Not all outcome measures presented as specified in methods. |
| Overall risk of bias | High |  |

| **Study** | Wu 2009 | |
| --- | --- | --- |
| **Domain being assessed** | VAS Pain | |
| **Risk of Bias Domain** | **Judgement** | **Support for judgement** |
| Randomisation process | Some concerns | Computer-generated random sequence.  Minimal information on allocation concealment.  No baseline differences between groups. |
| Deviation from the intended interventions | Low | Blinding through use of placebo. |
| Missing outcome data | Low | Outcome data available for all 60 randomised. |
| Measurement of the outcome | High | Outcome assessment highest pain score at unknown times.  Outcome assessors blinded. |
| Selection of the reported result | High | A single statistically significant outcome result reported. Multiple outcome measurements taken. |
| Overall risk of bias | High |  |

| **Study** | Yuksek 2003 | |
| --- | --- | --- |
| **Domain being assessed** | VAS Pain | |
| **Risk of Bias Domain** | **Judgement** | **Support for judgement** |
| Randomisation process | High | Minimal information on randomisation.  Likely lack of allocation concealment.  No baseline differences between groups. |
| Deviation from the intended interventions | Low | Blinding through use of placebo. |
| Missing outcome data | Low | Outcome data available for all 60 randomised. |
| Measurement of the outcome | Low | Outcome assessment done at same time point and frequency.  Outcome assessors blinded. |
| Selection of the reported result | High | Outcome not presented as specified in methods. |
| Overall risk of bias | High |  |
