# Supplementary material for: What is the impact of dexamethasone on postoperative pain in adults undergoing general anaesthesia for elective abdominal surgery: a systematic review and meta-analysis
Source: Perioper Med (Lond). 2022 Mar 24;11:13. doi: 10.1186/s13741-022-00243-6 (PMC8942613; doi:10.1186/s13741-022-00243-6)
Supplement: Supplementary file 5 — Additional file 5: Supplementary Figure 1 and Supplementary Figure 2. Summary risk of bias chart and weighted risk of bias chart. [file 13741_2022_243_MOESM5_ESM.docx]

Supplementary Figure 1 Summary risk of bias chart


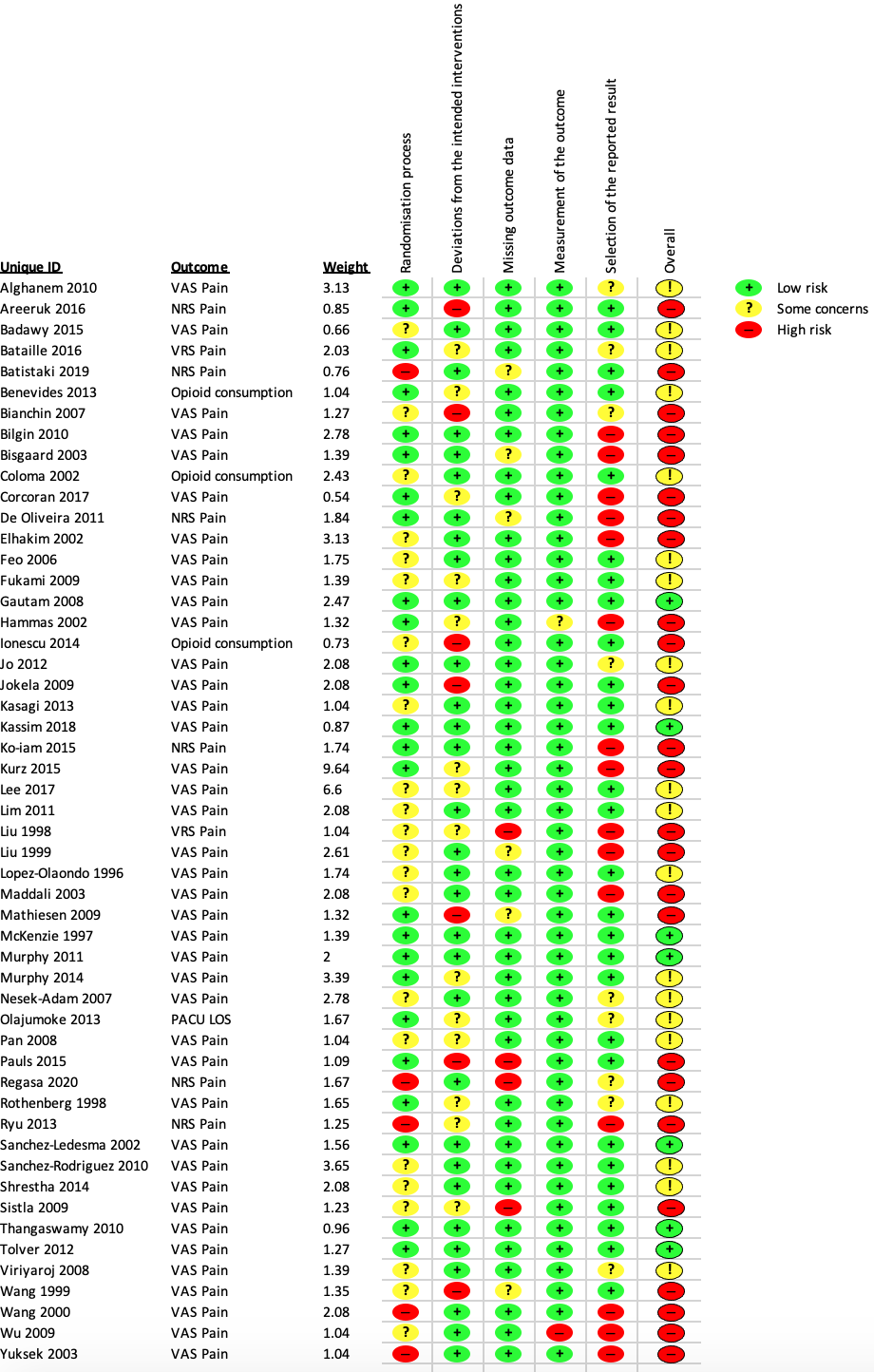


Supplementary Figure 2 Weighted risk of bias chart
