# Supplementary material for: What is the impact of dexamethasone on postoperative pain in adults undergoing general anaesthesia for elective abdominal surgery: a systematic review and meta-analysis
Source: Perioper Med (Lond). 2022 Mar 24;11:13. doi: 10.1186/s13741-022-00243-6 (PMC8942613; doi:10.1186/s13741-022-00243-6)

Supplementary Figure 3 Funnel plot for total postoperative opioid requirements.


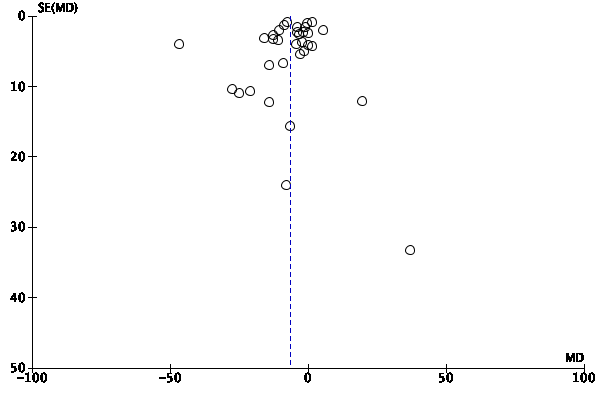


Supplementary Figure 4 Funnel plot for early (≤4 hours) VAS pain scores at rest.


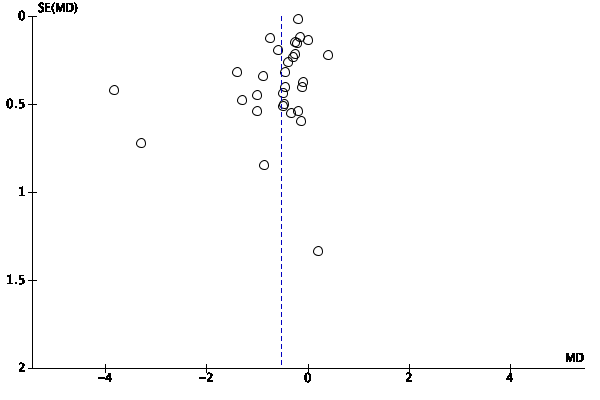

Supplement: Supplementary file 6 — Additional file 6: Supplementary Figure 3. Funnel plot for total postoperative opioid requirements. [file 13741_2022_243_MOESM6_ESM.docx]
