# Supplementary material for: What is the impact of dexamethasone on postoperative pain in adults undergoing general anaesthesia for elective abdominal surgery: a systematic review and meta-analysis
Source: Perioper Med (Lond). 2022 Mar 24;11:13. doi: 10.1186/s13741-022-00243-6 (PMC8942613; doi:10.1186/s13741-022-00243-6)

**Additional File 7**

**ADDITIONAL FOREST PLOTS**

FOREST PLOTS FOR PAIN SCORES

Forest plot for early (≤4 hours) VAS pain scores on movement.


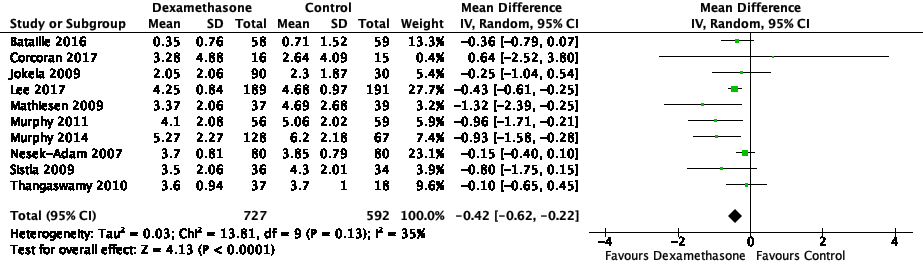


Forest plot for intermediate (4-24 hours) VAS pain scores at rest.


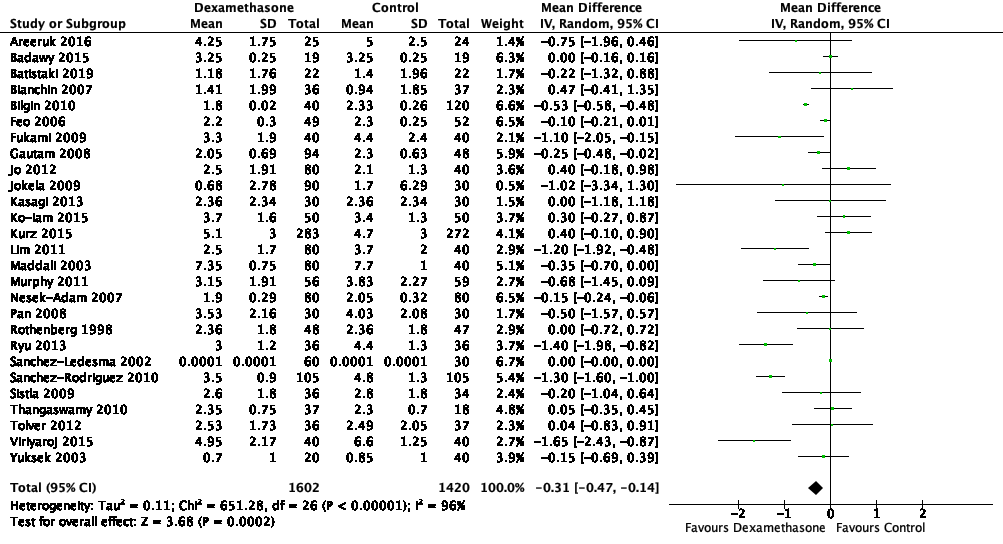


Forest plot for intermediate (4-24 hours) VAS pain scores on movement.


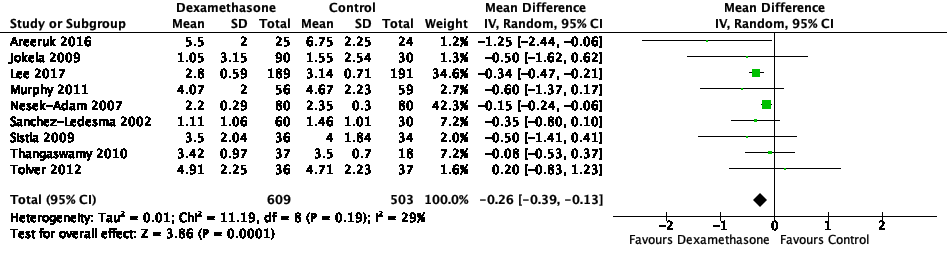


Forest plot for late (≥24 hours) VAS pain scores at rest.


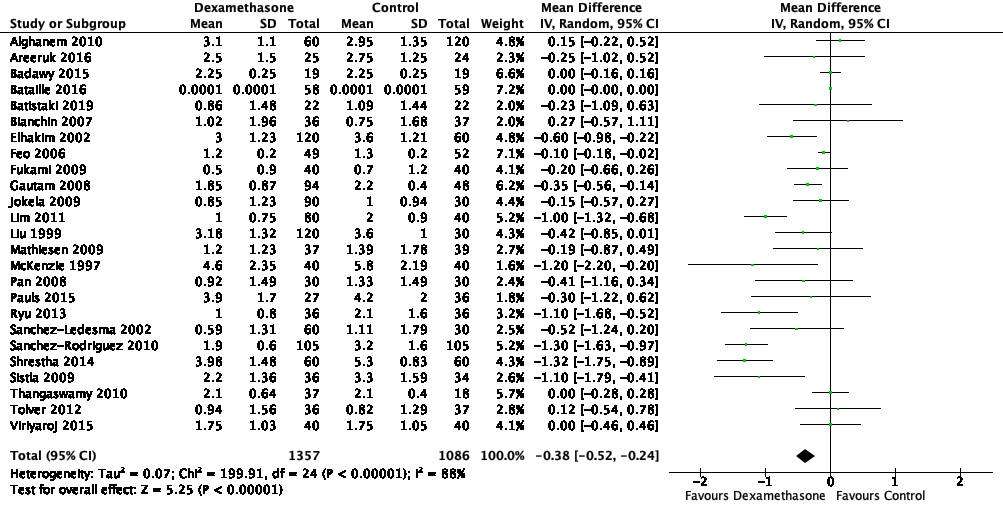


Forest plot for late (≥24 hours) VAS pain scores on movement.


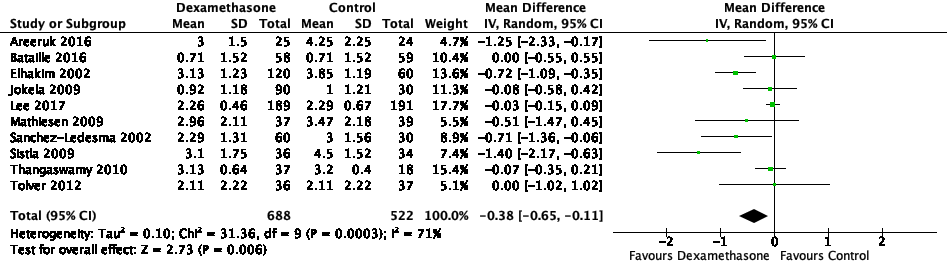


Forest plot for time to first analgesia in minutes.


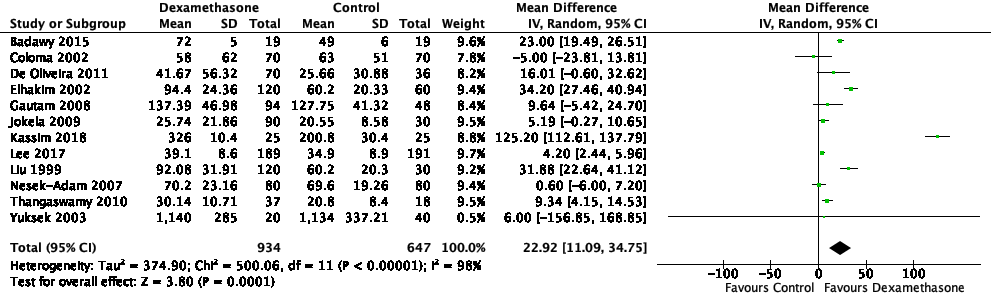


Forest plot for time to PACU discharge in minutes.


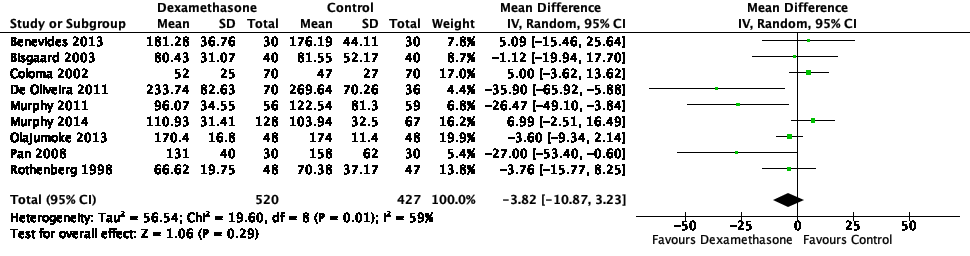


FOREST PLOTS FOR DOSING SUBGROUP ANALYSIS

Forest plot for dexamethasone dosing for early (≤4 hours) VAS pain scores at rest.


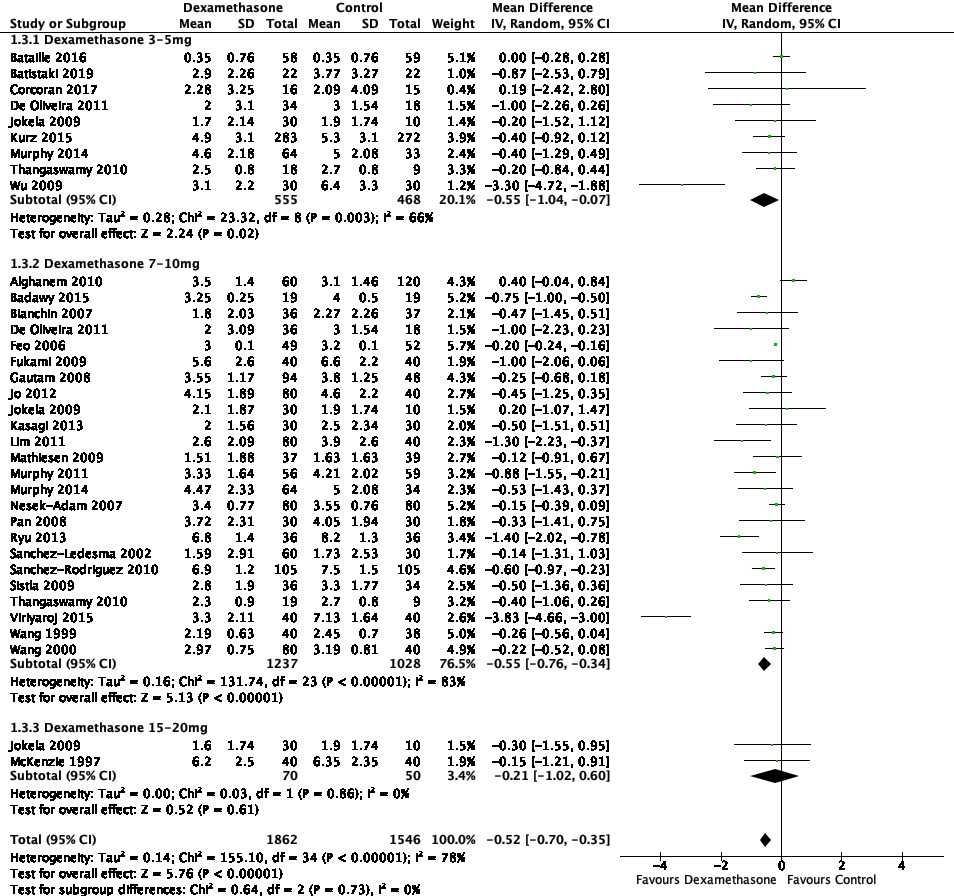


Forest plot for dexamethasone dosing for early (≤4 hours) VAS pain scores on movement.


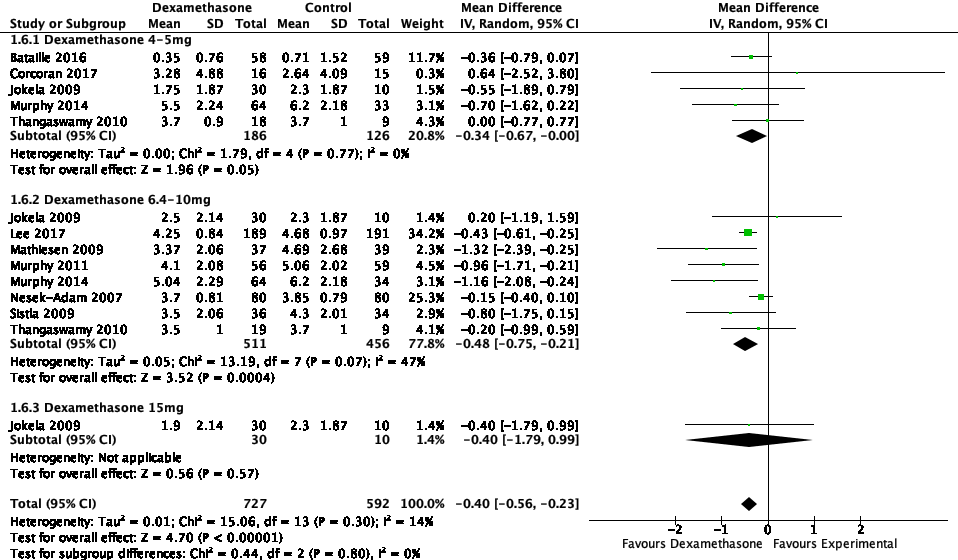


Forest plot for dexamethasone dosing intermediate (4-24 hours) VAS pain scores at rest.


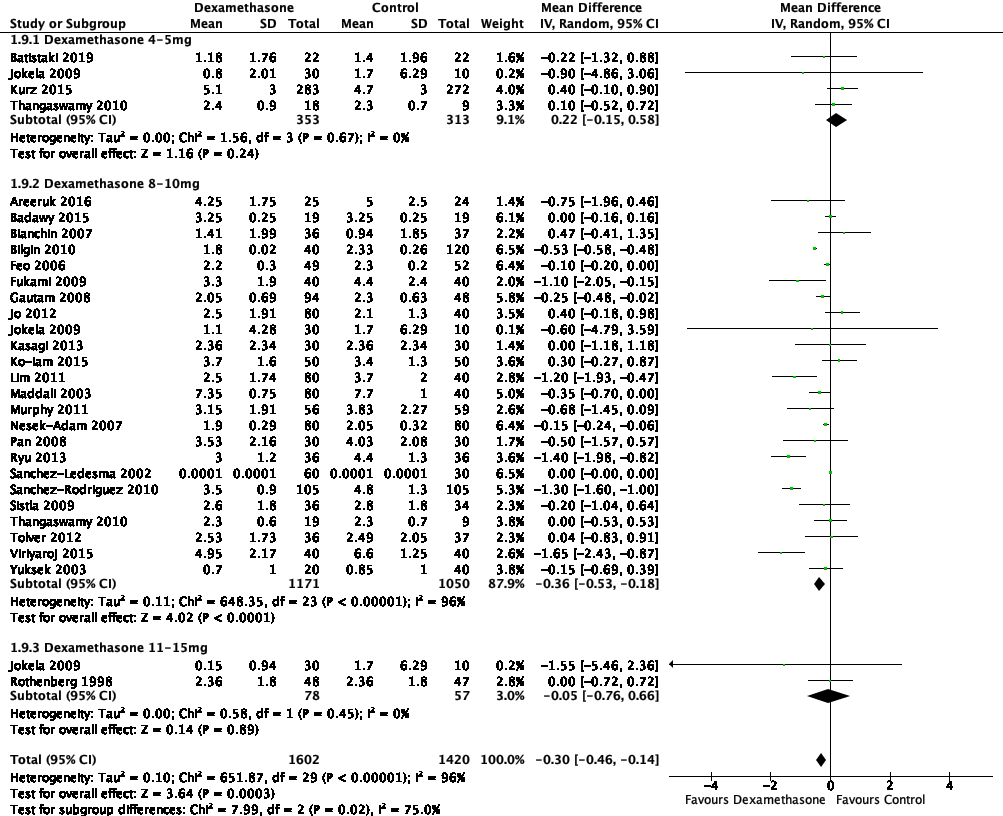


Forest plot for dexamethasone dosing intermediate (4-24 hours) VAS pain scores on movement.


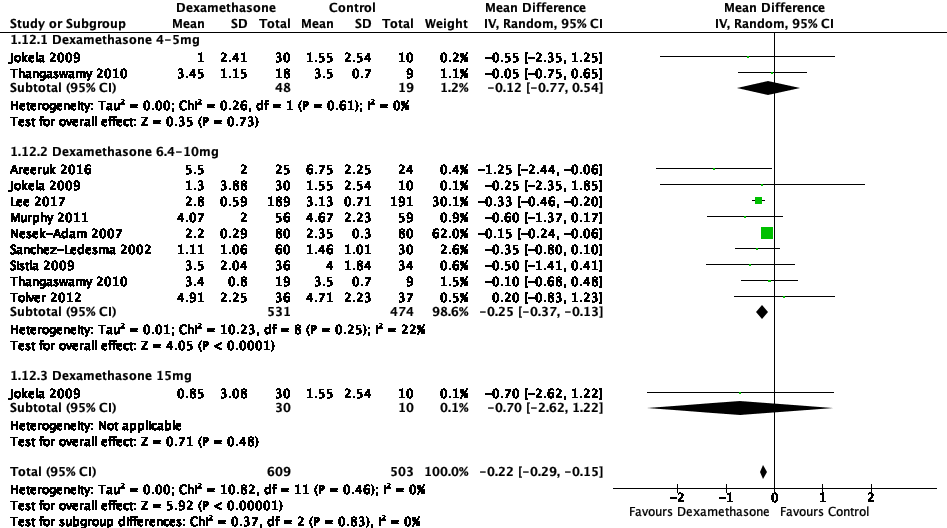


Forest plot for dexamethasone dosing late (≥24 hours) VAS pain scores on movement.


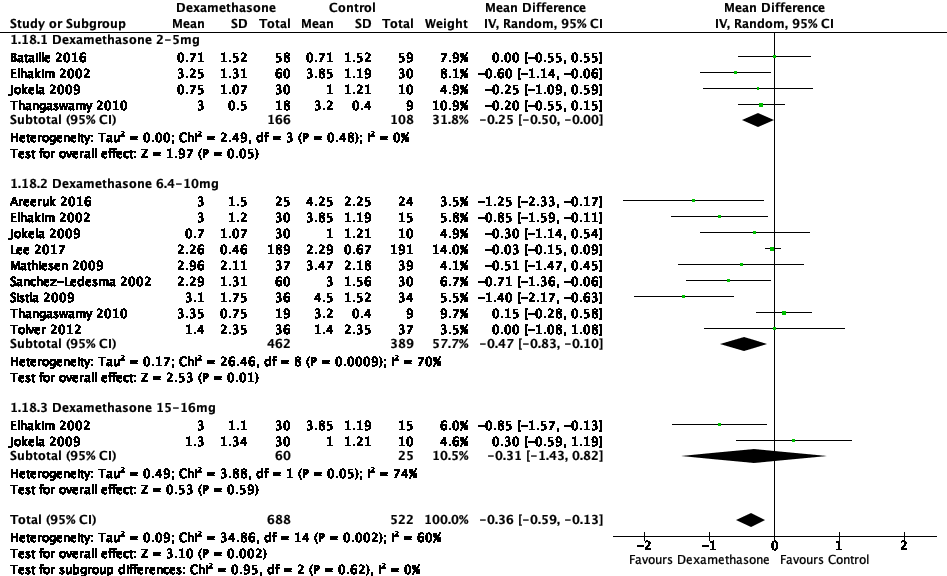


Forest plot for dexamethasone dosing for time to first analgesia.


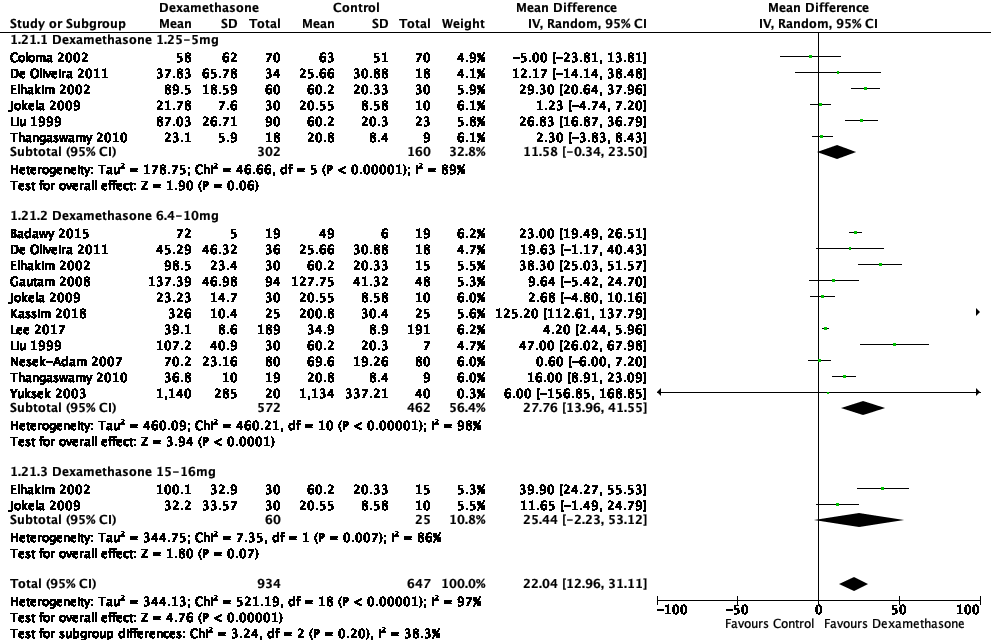


Forest plot for dexamethasone dosing for total postoperative opioid use in mg of oral morphine equivalents.


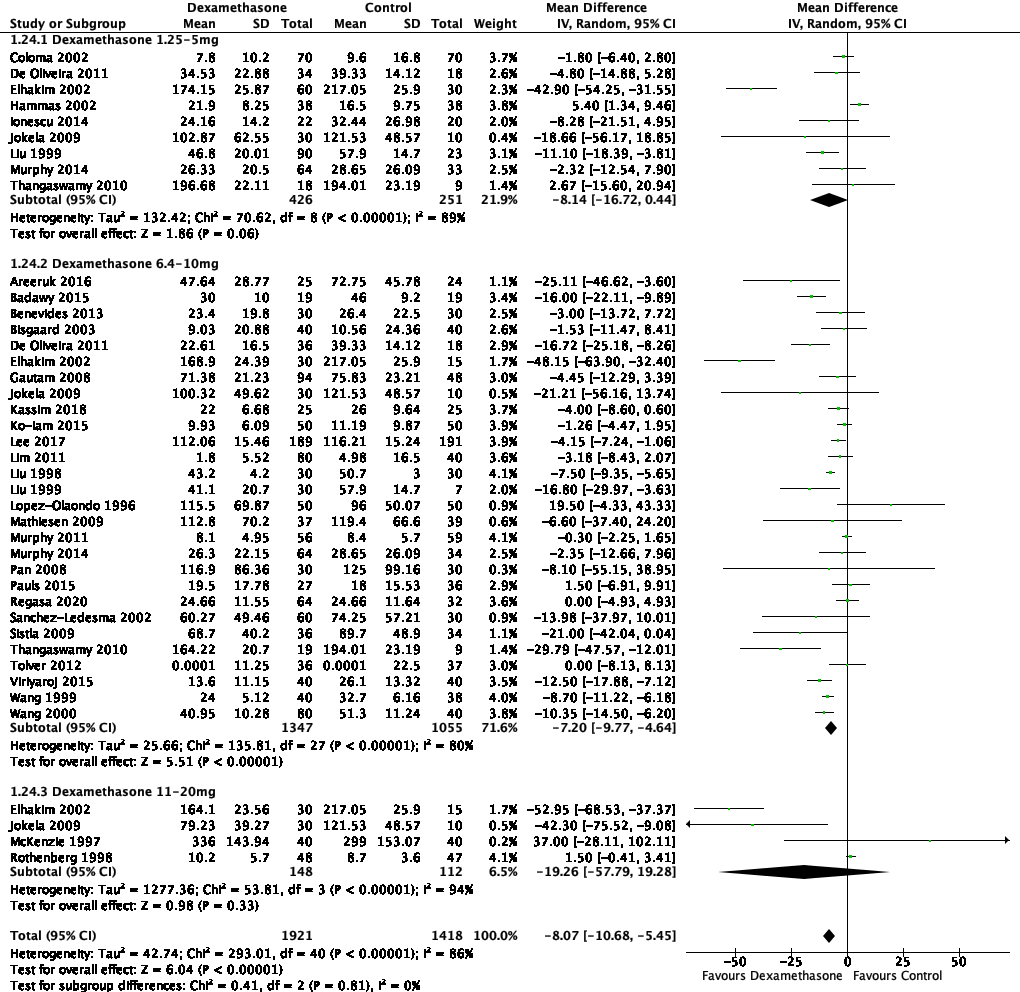


Forest plot for dexamethasone dosing for time to PACU discharge.


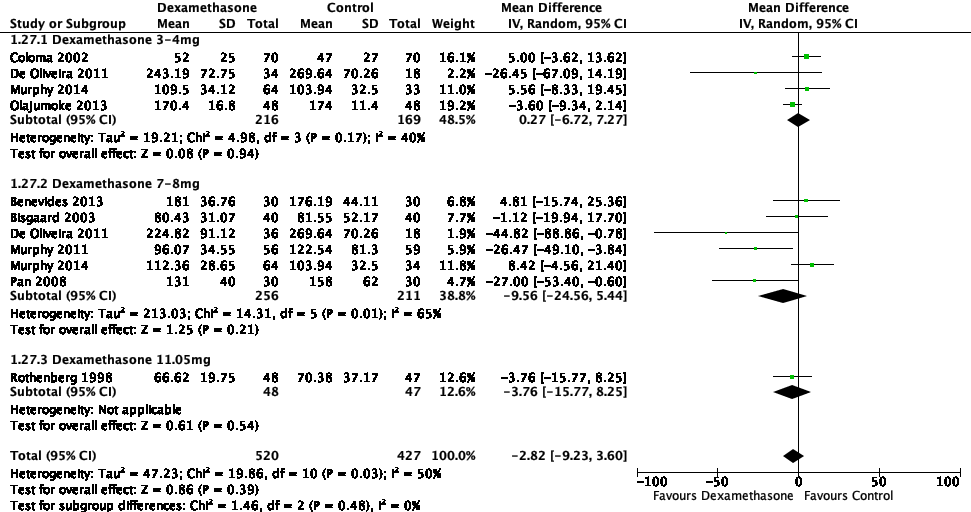


FOREST PLOTS FOR TIMING SUBGROUP ANALYSIS

Forest plot for dexamethasone timing for early (≤4 hours) VAS pain scores at rest.


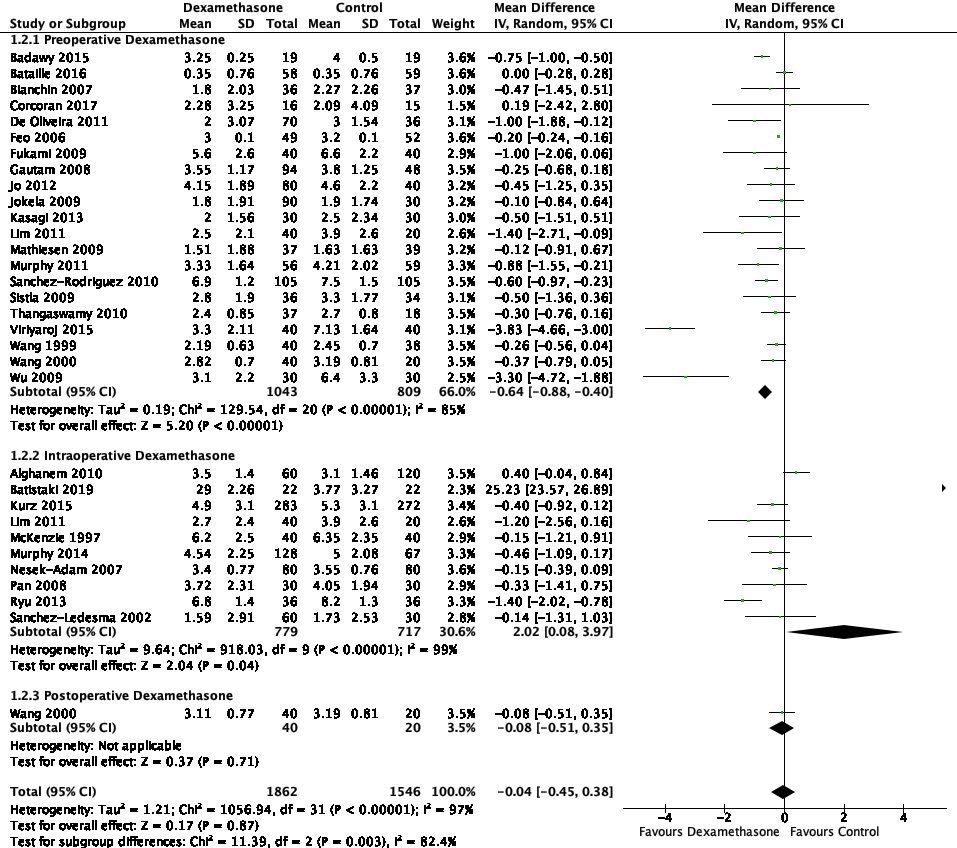


Forest plot for dexamethasone timing for early (≤4 hours) VAS pain scores on movement.


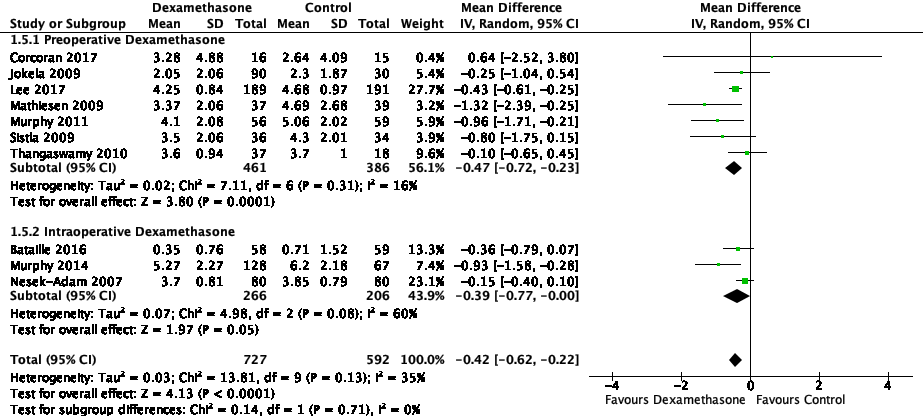


Forest plot for dexamethasone timing for intermediate (4-24 hours) VAS pain scores at rest.


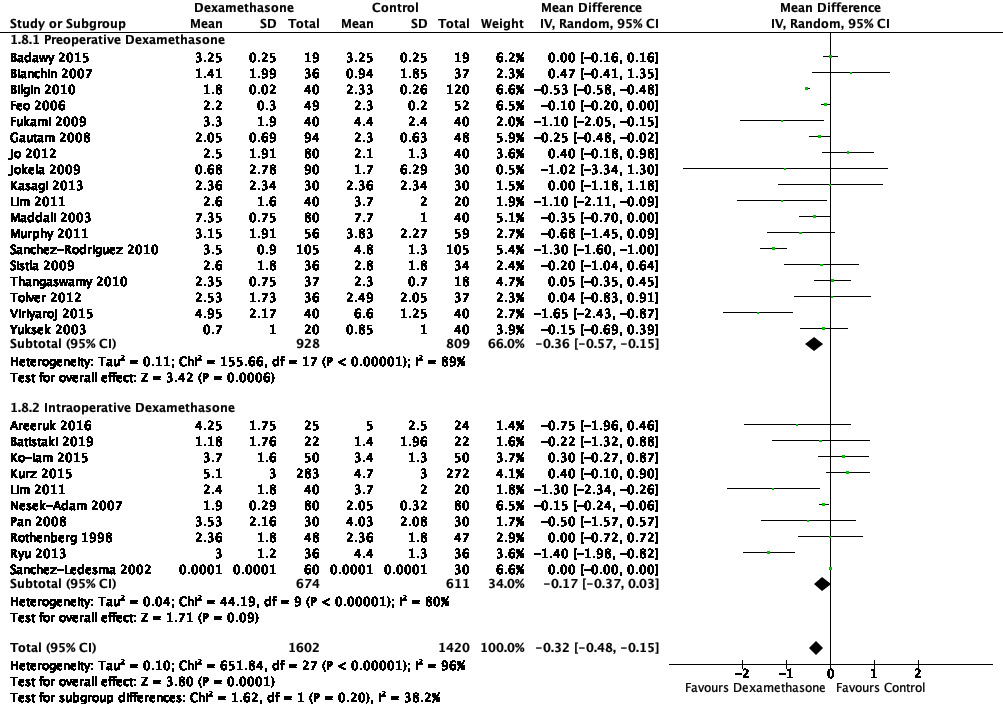


Forest plot for dexamethasone timing for intermediate (4-24 hours) VAS pain scores on movement.


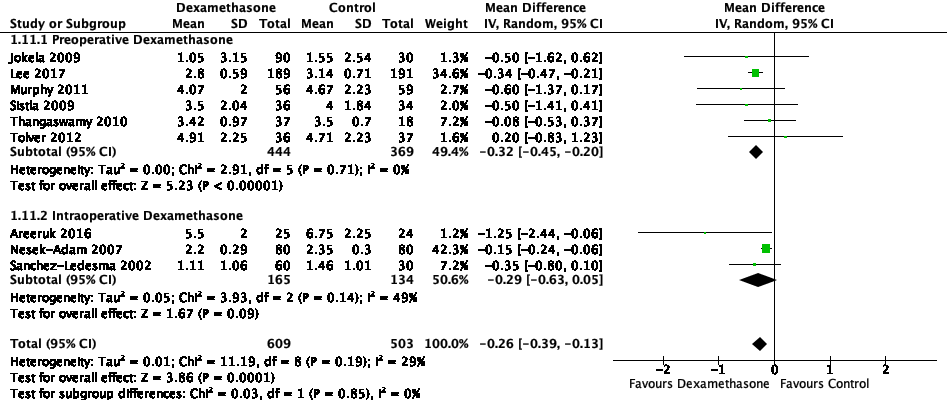


Forest plot for dexamethasone timing for late (≥24 hours) VAS pain scores at rest.


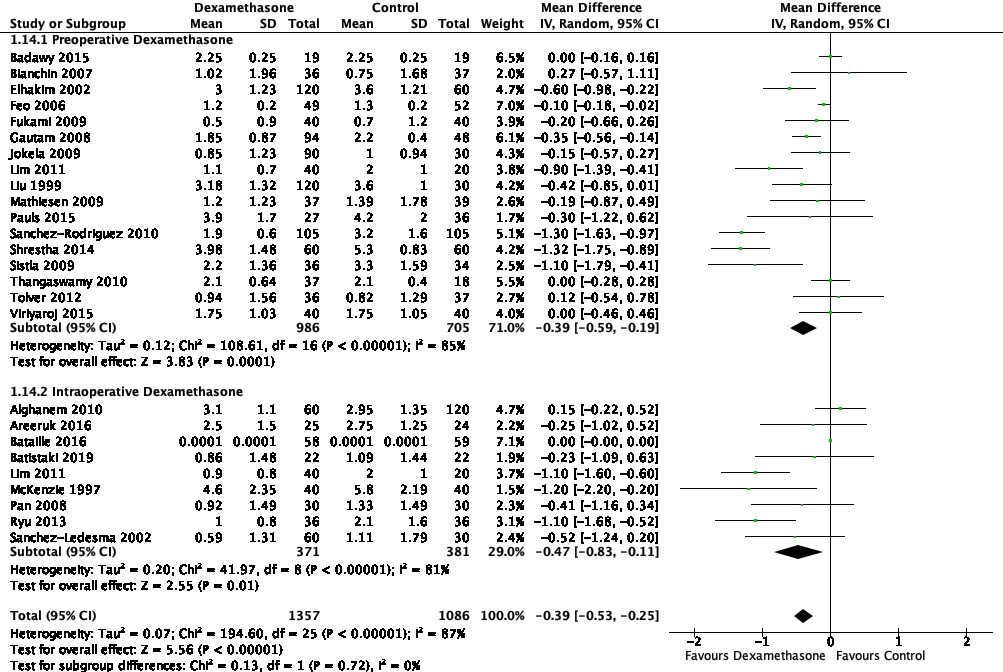


Forest plot for dexamethasone timing for late (≥24 hours) VAS pain scores on movement.


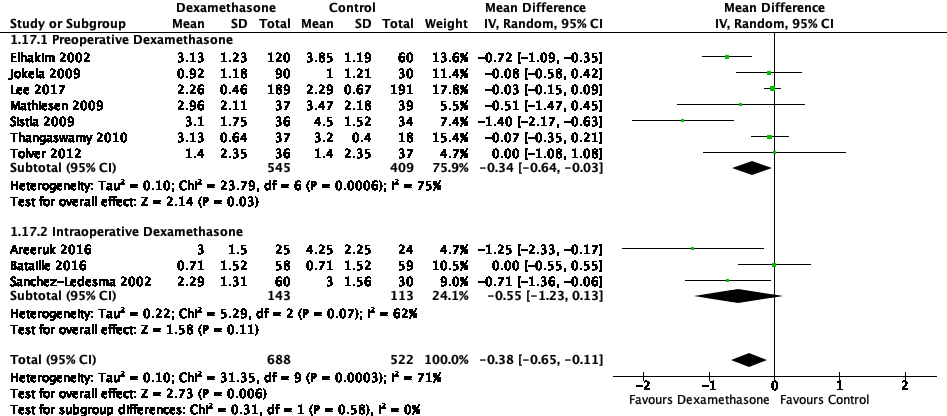


Forest plot for dexamethasone timing for total postoperative opioid use in mg of oral morphine equivalents.


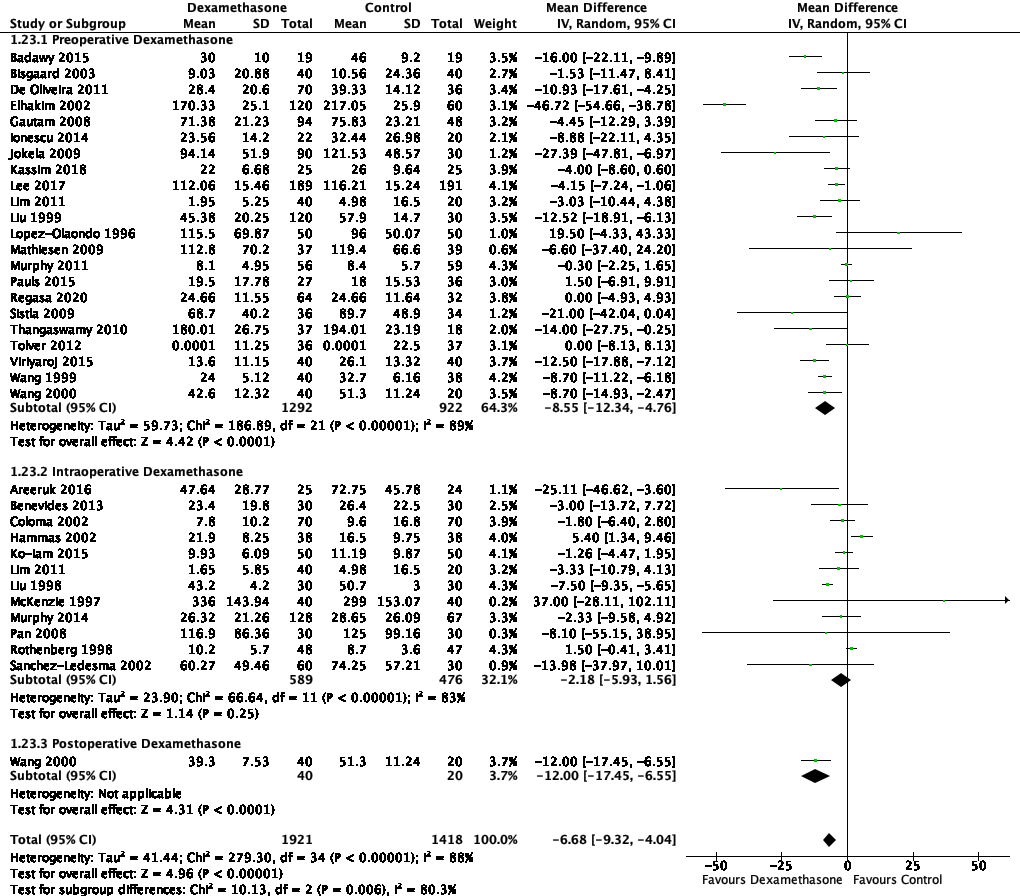


Forest plot for dexamethasone timing for time to first analgesia in minutes.


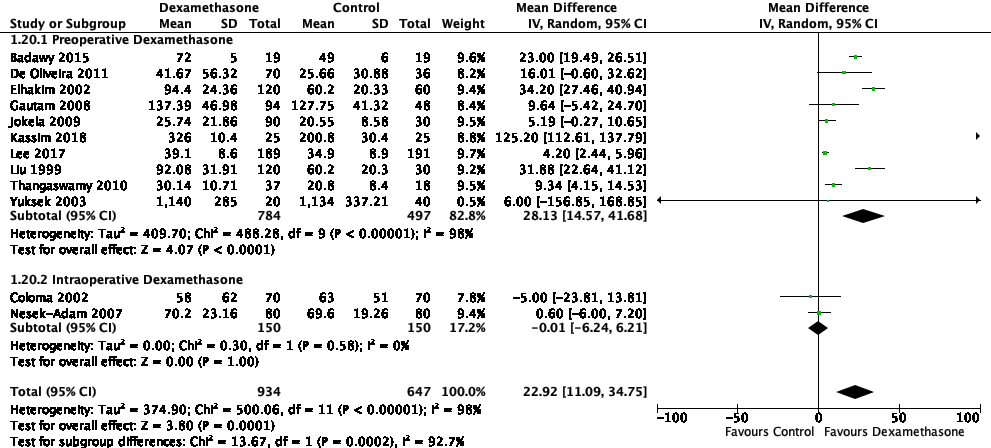


Forest plot for dexamethasone timing for time to PACU discharge in minutes.


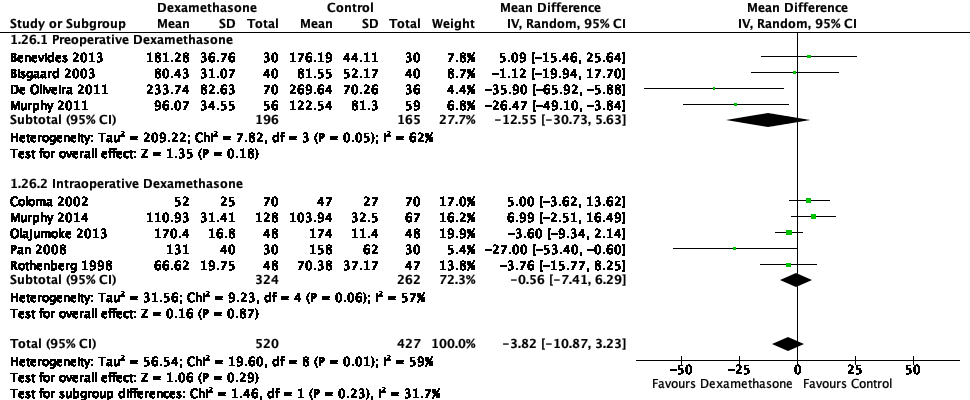

Supplement: Supplementary file 7 — Additional file 7. Additional forest plots [file 13741_2022_243_MOESM7_ESM.docx]
